# Supplementary material for: Cooperative microbial interactions drive spatial segregation in porous environments
Source: Nat Commun. 2023 Jul 15;14:4226. doi: 10.1038/s41467-023-39991-4 (PMC10349867; doi:10.1038/s41467-023-39991-4)
Supplement: Supplementary file 1 — Supplementary Information [file 41467_2023_39991_MOESM1_ESM.pdf]

Supplementary information

Cooperative microbial interactions drive spatial segregation in porous environments

Yichao Wu[1,#], Chengxia Fu[1,#], Caroline L. Peacock[2], Søren J. Sørensen[3], M.A. Redmile-Gordon[4], Keqing Xiao[5,2], Chunhui Gao[1], Jun Liu[1], Qiaoyun Huang[1], Zixue Li[6], Peiyi Song[6], Yongguan Zhu[5,7], Jizhong Zhou[8,9,10,11], Peng Cai*[,1]

1 State Key Laboratory of Agricultural Microbiology, College of Resources and Environment, Huazhong Agricultural University, Wuhan, China

2 School of Earth and Environment, University of Leeds, Leeds LS2 9JT, United Kingdom

3 Section of Microbiology, Department of Biology, University of Copenhagen, Copenhagen, Denmark

4 Department of Environmental Horticulture, Royal Horticultural Society, Wisley, Surrey, GU23 6QB, United Kingdom

5 State Key Laboratory of Urban and Regional Ecology, Research Center for Eco-Environmental Sciences, Chinese Academy of Sciences, Beijing, China.

6 School of Physics, Huazhong University of Science and Technology, Wuhan, China.

7 Key Laboratory of Urban Environment and Health, Institute of Urban Environment, Chinese Academy of Sciences, Xiamen, China

8 Institute for Environmental Genomics and Department of Microbiology and Plant Biology, University of Oklahoma, Norman, USA

9 State Key Joint Laboratory of Environment Simulation and Pollution Control, School of Environment, Tsinghua University, Beijing, China

10 Earth and Environmental Sciences, Lawrence Berkeley National Laboratory, Berkeley, USA

11 School of Civil Engineering and Environmental Sciences, University of Oklahoma, Norman, USA

[#]The authors contributed equally to this work.

*Corresponding author: Peng Cai, cp@mail.hzau.edu.cn

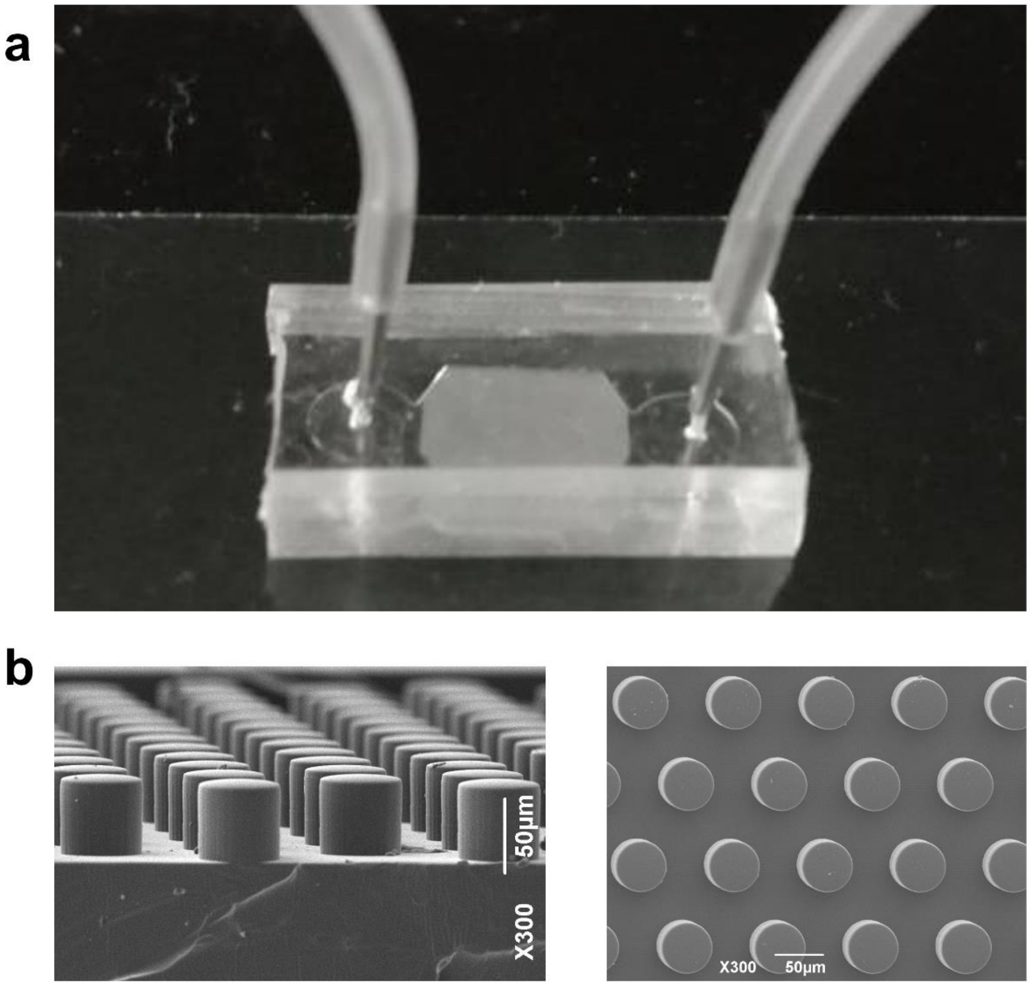

Supplementary Fig. 1. The photograph (a) and SEM images (b) of the microfluidic chamber consisting of a matrix of pillars. The diameter and height of pillars are 50 μm. The SEM characterization was performed on three independent microfluidic chips and consistent results were obtained.

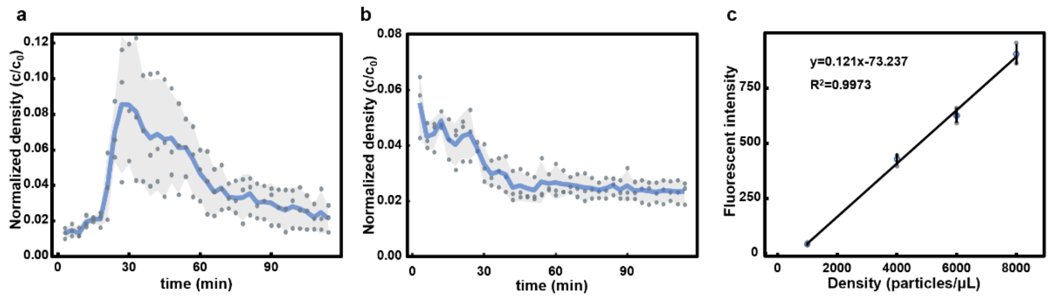

Supplementary Fig. 2. The breakthrough curve of microsphere in the microfluidic chamber without (a) and with 48-h-old biofilm (b). The microsphere density in the effluent was normalized by the density of the influent. The solid line represents the average density of microspheres in the effluent and the shaded areas indicate the standard deviation of three independent replicates. The linear correlation between the fluorescent intensity and the microsphere density (c). Three independent replicates were conducted for each microsphere density. Data are presented as mean values ± standard deviation.

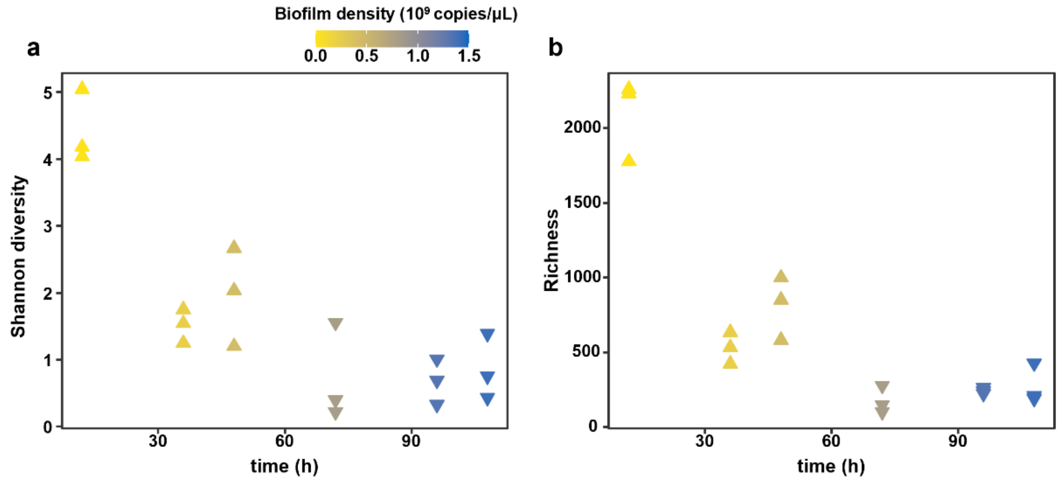

Supplementary Fig. 3. The decreased diversity (a) and richness (b) during early biofilm development.

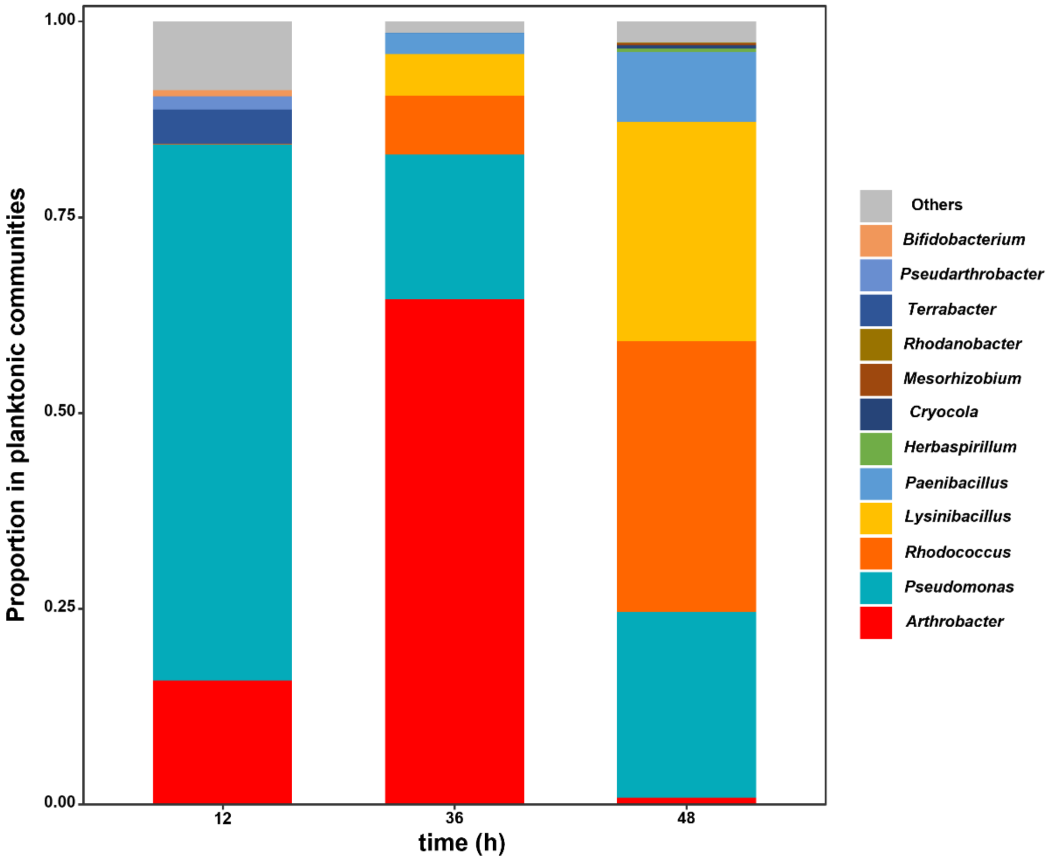

Supplementary Fig. 4. The composition of planktonic communities at the genus level (n = 3 chips).

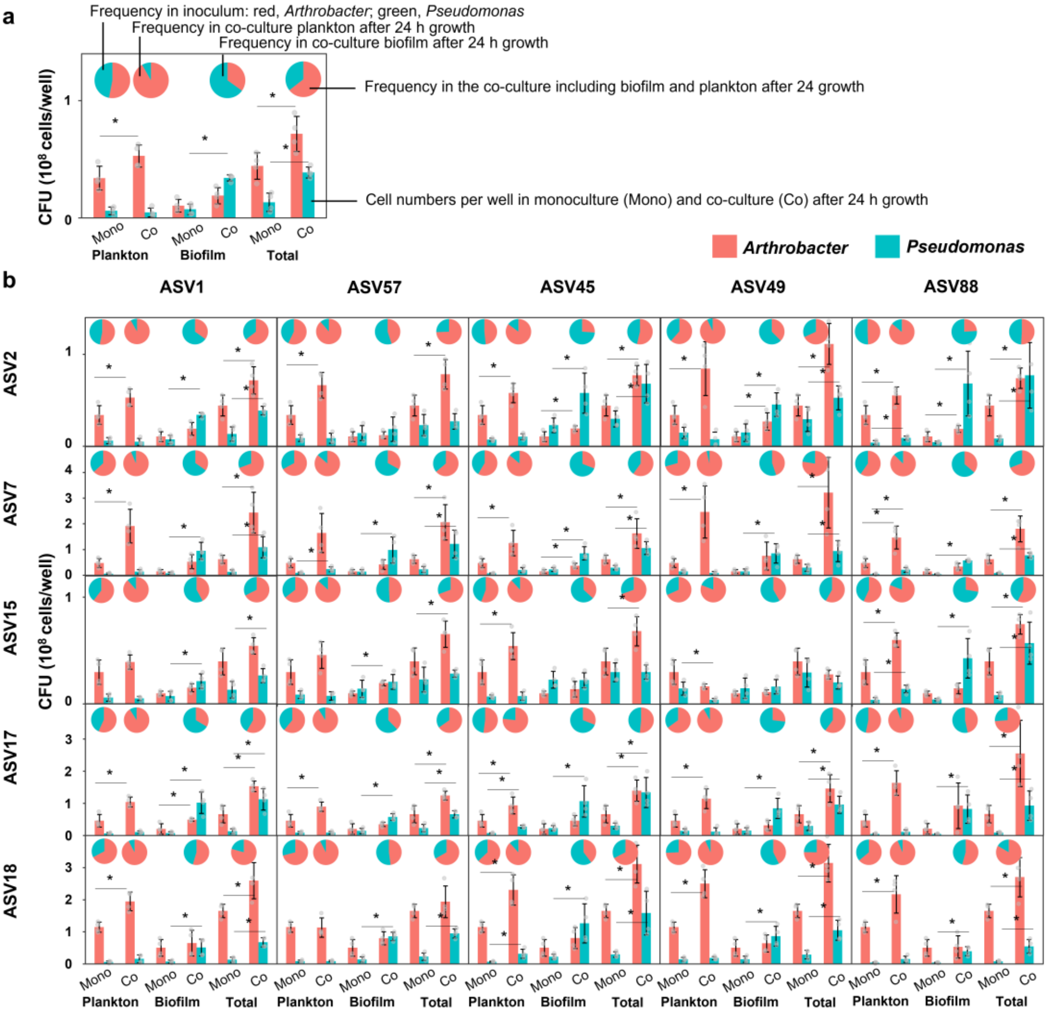

Supplementary Fig. 5. The abundance of *Arthrobacter* and *Pseudomonas* in co-culture. 5 *Arthrobacter* isolates (ASV2, ASV7, ASV15, ASV17 and ASV18) were mixed with 5 *Pseudomonas* isolates (ASV1, ASV57, ASV45, ASV49 and ASV88) in pairwise combinations. After 24 h growth, the cell numbers were determined by genus-specific qPCR. As shown in Supplementary Fig. 5a, the pie charts from left to right are the frequencies of the two genotypes in the inoculum, co-culture plankton, co-culture biofilm and the total community, respectively. The columns from left to right represent the cell numbers of the two genotypes in monoculture plankton, co-culture plankton, monoculture biofilm, co-culture biofilm and the total cell numbers for monoculture and co-culture. Data are presented as mean values ± standard deviation. The

asterisk indicates significant difference in the cell numbers of co-culture and monoculture ($p < 0.05$, two-tailed Student's *t*-test). For exact *p* values, see Supplementary Table 2. Source data are provided as a Source Data file.

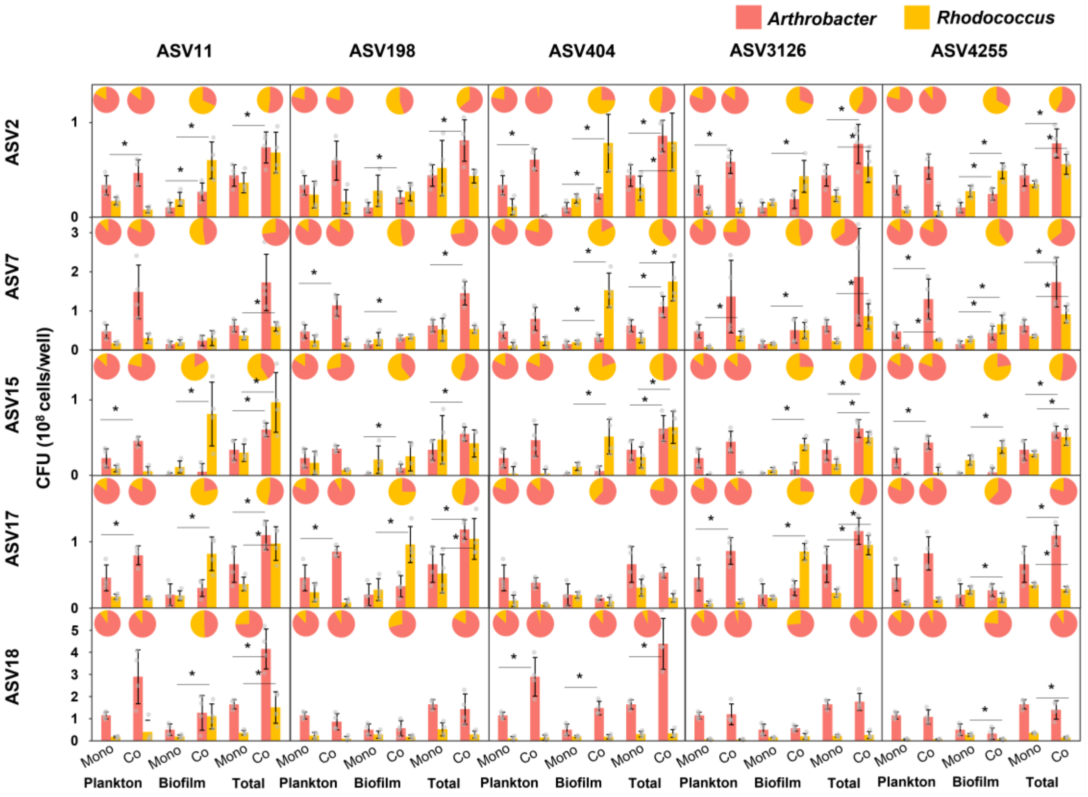

Supplementary Fig. 6. The abundance of *Arthrobacter* and *Rhodococcus* in co-culture. 5 *Arthrobacter* isolates (ASV2, ASV7, ASV15, ASV17 and ASV18) were mixed with 5 *Rhodococcus* isolates (ASV11, ASV198, ASV404, ASV3126 and ASV4255) in pairwise combinations. After 24 h growth, the cell numbers were determined by genus-specific qPCR. Data are presented as mean values ± standard deviation. The asterisk indicates significant difference in the cell numbers of co-culture and monoculture ($p < 0.05$, two-tailed Student's t-test). For exact p values, see Supplementary Table 3. Source data are provided as a Source Data file.

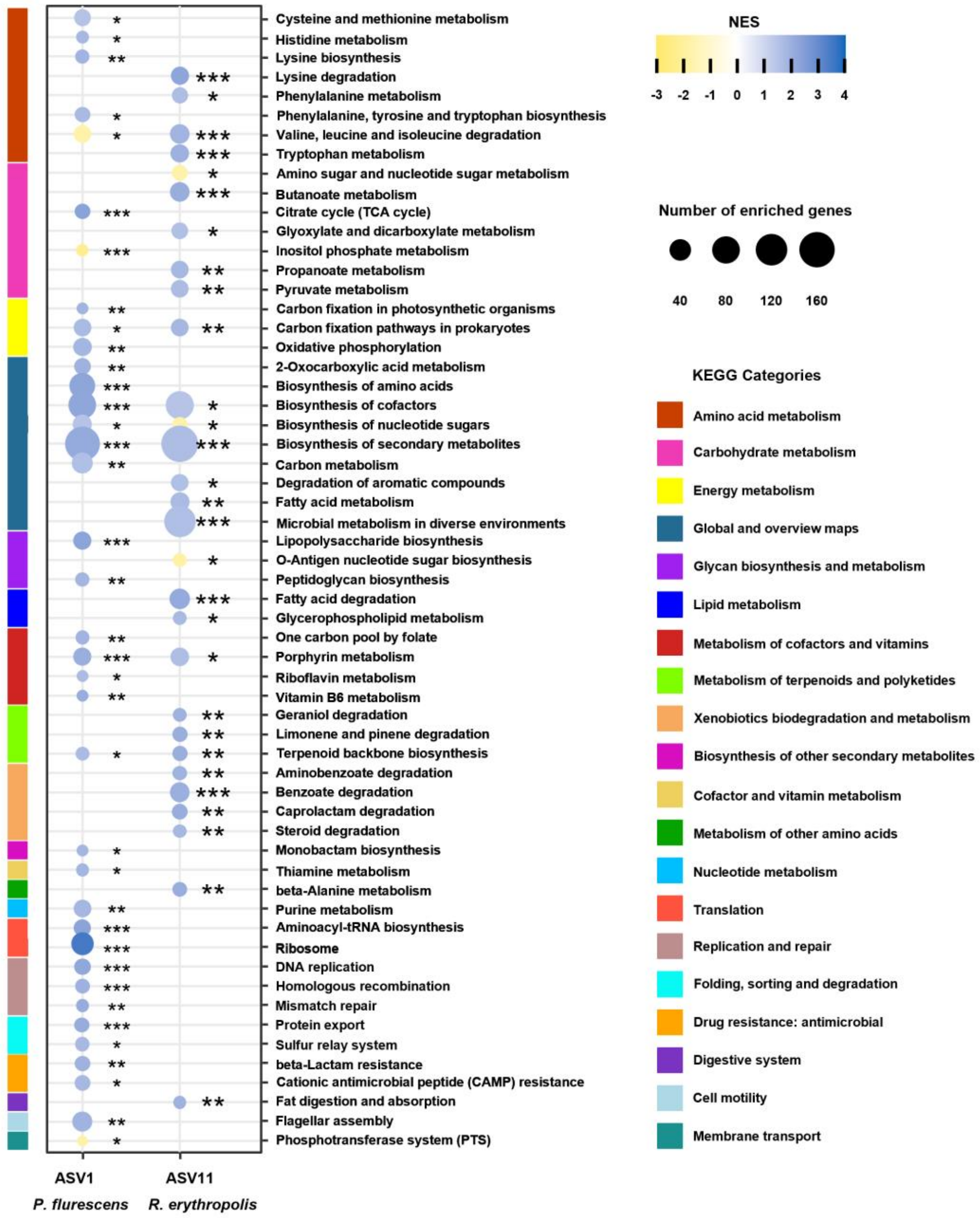

Supplementary Fig. 7. Gene set enrichment analysis (GSEA) reveals the KEGG pathways enriched in co-culture with ASV2 *A. ramosus*. GSEA ranked genes based on the $\log_2$ fold change between co-culture and monoculture by which most significantly upregulated and downregulated genes in co-culture were at the top and bottom of the gene list, respectively. A significant positive normalized enrichment score (NES) represents that the genes in the KEGG pathway are enriched at the top of the ordered gene list, while negative NES indicates that the genes in the pathway are overrepresented

at the bottom of the ranked list. The size of the circle represents the number of core enriched genes which contributed to NES. The nominal *P* value for the observed NES was determined relative to null distribution. *, $p < 0.05$; **, $p < 0.01$; ***, $p < 0.001$. Source data are provided as a Source Data file.

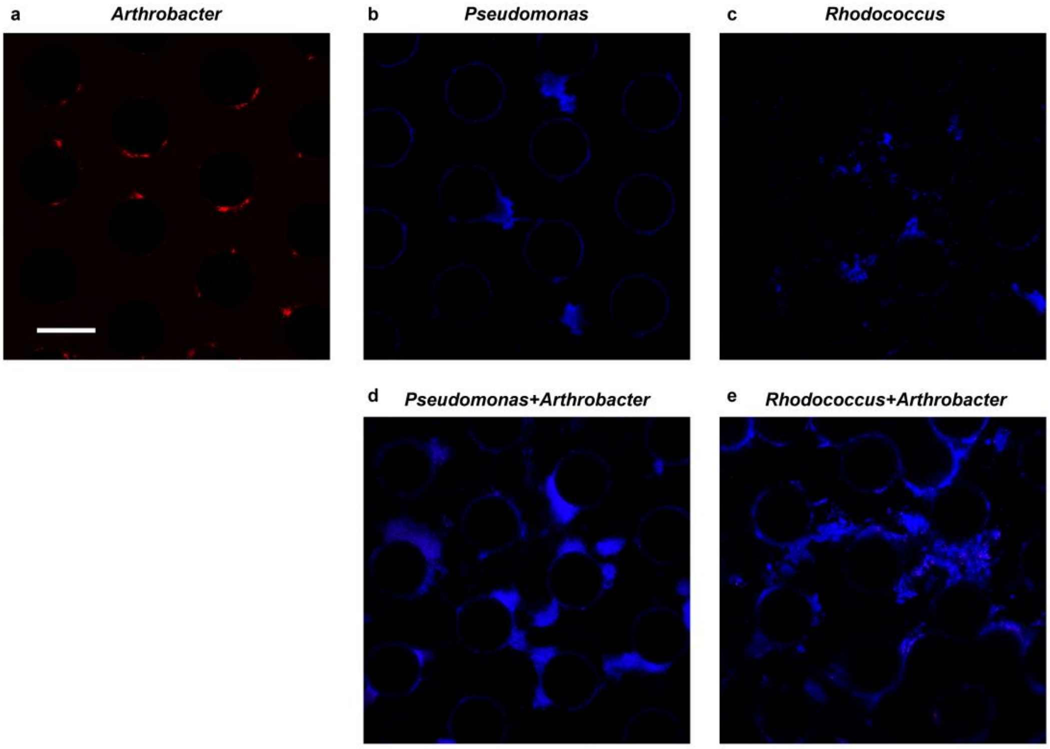

Supplementary Fig. 8. Co-cultivation with ASV2 *A. ramosus* enhances biofilm formation. Monoculture biofilm of ASV2 *A. ramosus* (a), ASV1 *P. fluorescens* (b) and ASV11 *R. erythropolis* (c). The dual-species biofilms formed by *Pseudomonas-Arthrobacter* (d) and *Rhodococcus-Arthrobacter* (e). Biofilm cells are cultured in microfluidic chips for 48 hours and then stained with DAPI (blue). *Arthrobacter* in biofilm is hybridized with ART179 (Alexa546, red). Scale bar represents 50 μm. Each combination was repeated in three independent microfluidic chips with similar results.

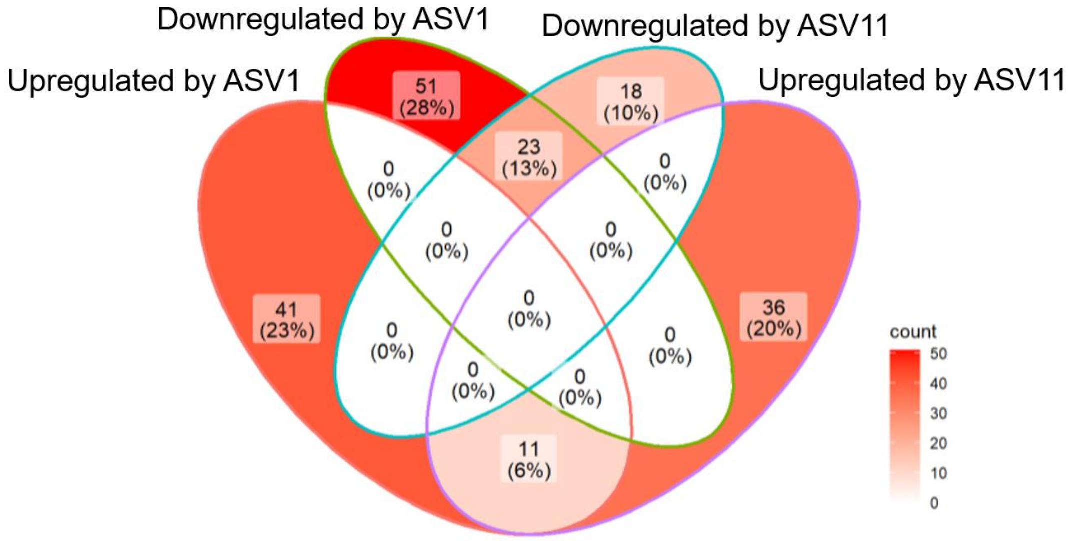

Supplementary Fig. 9. Venn diagram of differentially expressed genes in ASV2 *A. ramosus* in co-culture compared to monoculture. Source data are provided as a Source Data file.

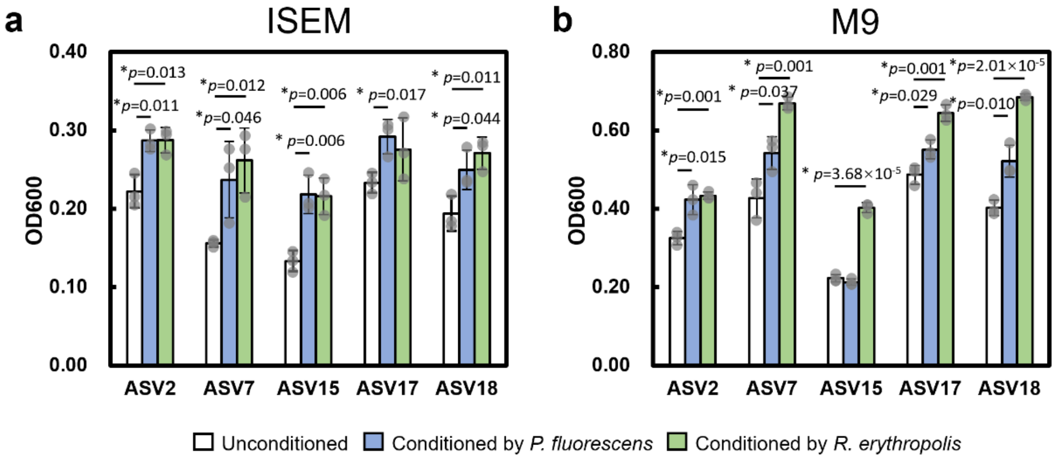

Supplementary Fig. 10. The enhanced growth of *Arthrobacter* strains in ISEM (a) and M9 minimal medium (b) conditioned by ASV1 *P. fluorescens* and ASV11 *R. erythropolis*. OD_{600} was measured after 48 h cultivation. The columns represent the average values of three independent replicates (shown as grey dots) and the error bars indicate the standard deviation. The asterisk indicates significant difference ($p < 0.05$, two-tailed Student's *t*-test).

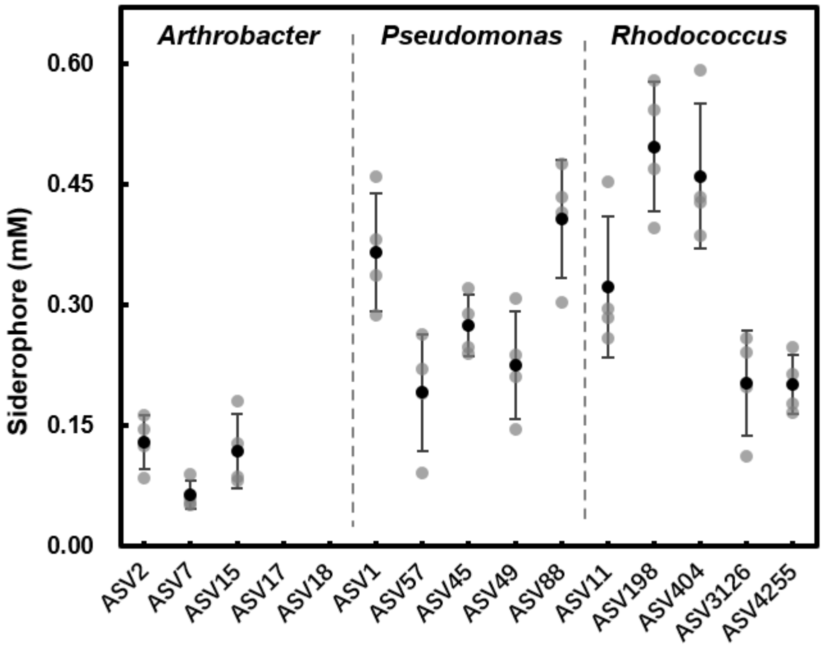

Supplementary Fig. 11. The siderophore concentration in the culture grown in ISEM for 48 h. The black dots represent the average values of four independent replicates (shown as grey dots) and the error bars indicate the standard deviation. The siderophore concentrations in the cultures of ASV17 and ASV18 were below the detection limit (0.045 mM).

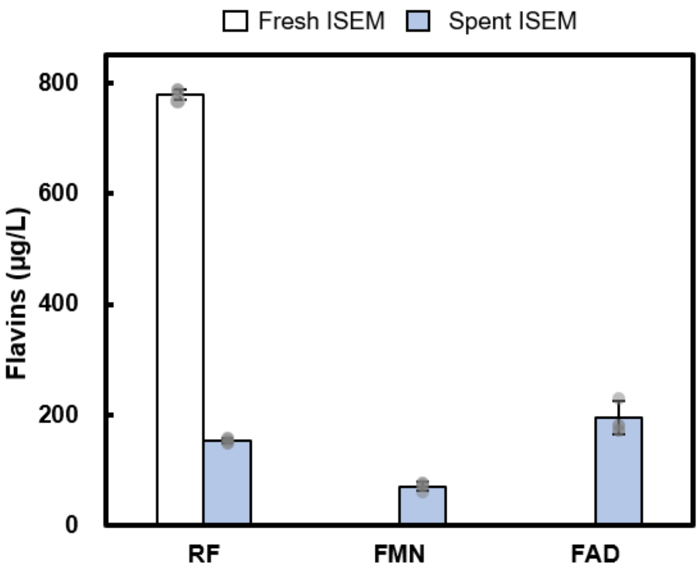

Supplementary Fig. 12. The flavin concentrations in fresh ISEM and the supernatant of ASV1 *P. fluorescens* after 48 h cultivation in ISEM (n = 3 biologically independent replicates). FMN and FAD were not detected in fresh ISEM. The detection limits for FMN and FAD were 50 μg/L. Data are presented as mean values ± standard deviation.

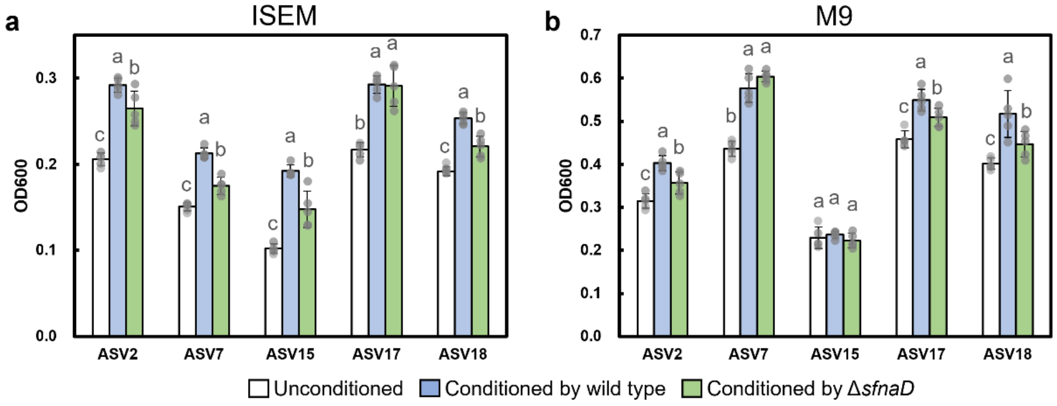

Supplementary Fig. 13. The growth of *Arthrobacter* strains in ISEM (a) and M9 minimal medium (b) conditioned by wild-type ASV1 *P. fluorescens* and the siderophore synthesis mutant (Δ*sfnaD*). OD_{600} was measured after 48 h cultivation. Four independent replicates were carried out. Data are presented as mean values ± standard deviation. Different letters indicate significant differences in the relative growth of individual strains under different conditions ($p < 0.05$, one-way ANOVA), whilst shared letters indicate no statistical difference. For exact *p* values, see Supplementary Table 4.

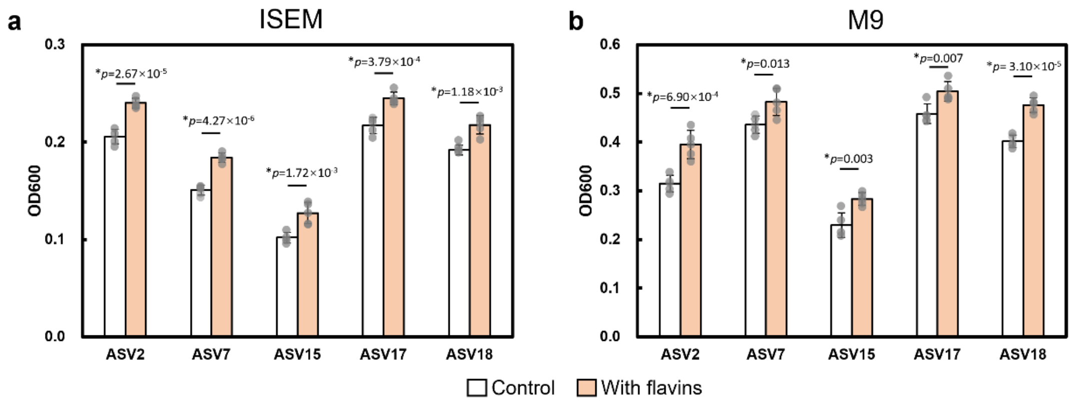

Supplementary Fig. 14. Flavins enhanced the growth of *Arthrobacter* strains in ISEM (a) and M9 minimal medium (b). 100 μg/L FAD and 35 μg/L FMN were added to both ISEM and M9 medium. The growth of *Arthrobacter* strains in fresh ISEM and M9 medium was used as the control group. OD_{600} was monitored after 48 h cultivation. Four independent replicates were carried out. Data are presented as mean values ± standard deviation. The asterisk indicates significant difference ($p < 0.05$, two-tailed Student's t-test).

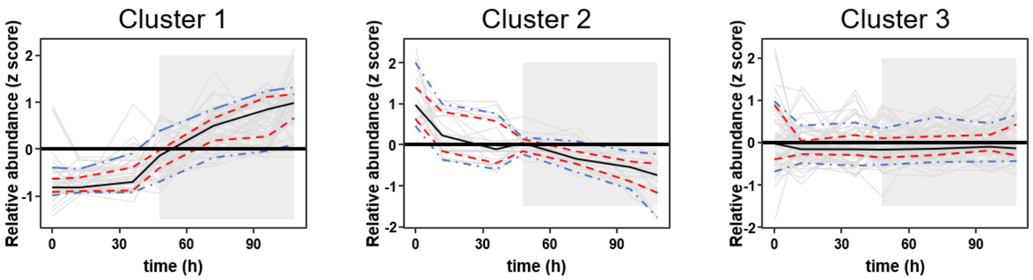

Supplementary Fig. 15. Changes in exometabolites reveal three unique patterns, including released (cluster 1), consumed (cluster 2) and the others (cluster 3). The abundance of each exometabolite (peak areas from LC-MS/MS) was z-score normalized across all samples. The solid line represents the median values in each cluster. The blue lines represent 10th and 90th percentiles and the red lines indicate 25th and 75th percentiles.

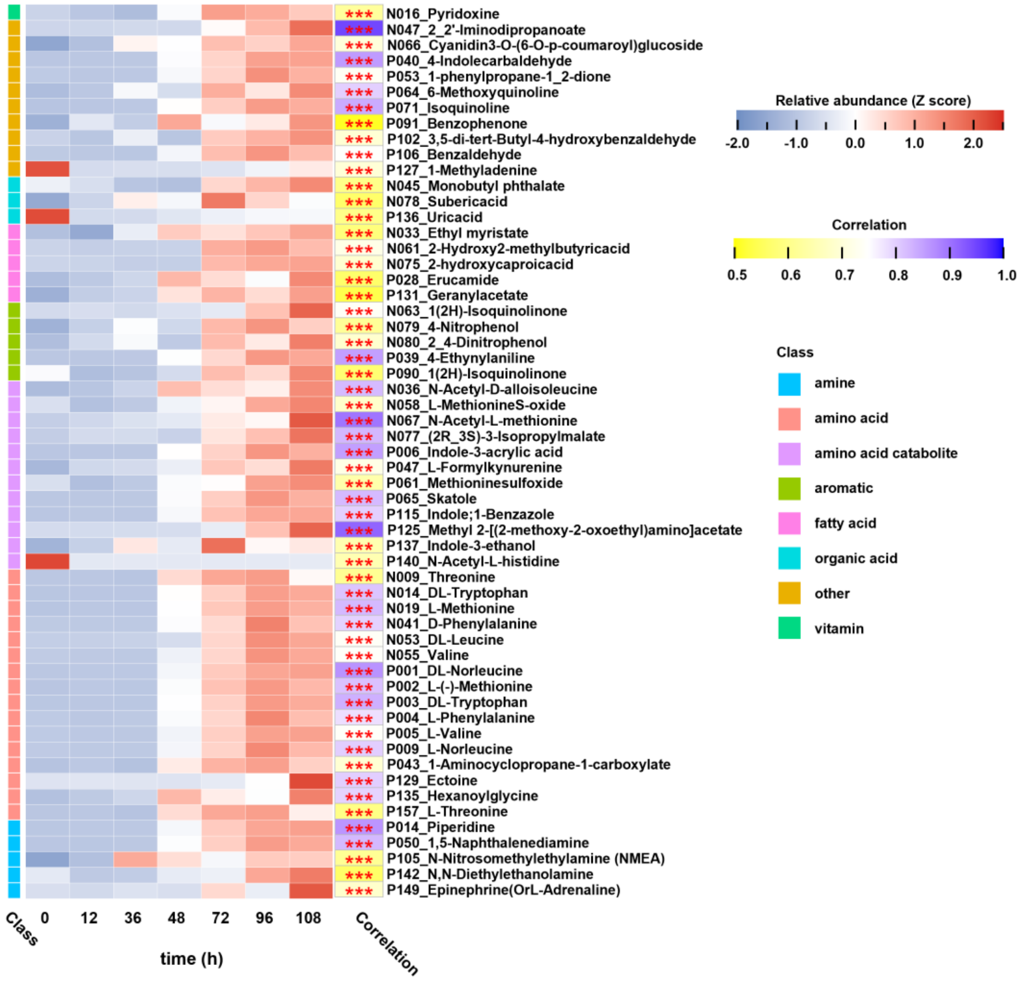

Supplementary Fig. 16. The dynamics of exometabolites in cluster 1 (n = 6 biological replicates for each time point). The first letter in the labels indicates the metabolite detected in positive (P) or negative (N) mode. Spearman correlation coefficients between metabolite abundance and the incubation period were determined. *, $p < 0.05$; **, $p < 0.01$; ***, $p < 0.001$. Source data are provided as a Source Data file.

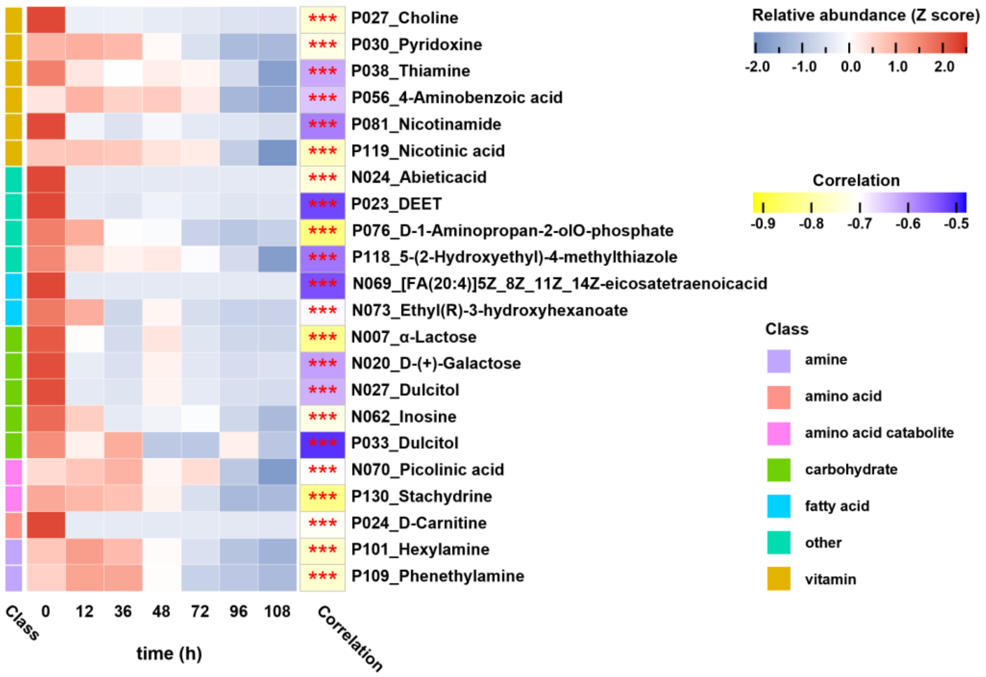

Supplementary Fig. 17. The dynamics of exometabolites in cluster 2 (n = 6 biological replicates for each time point). Spearman correlation coefficients between metabolite abundance and the incubation period were determined. *, $p < 0.05$; **, $p < 0.01$; ***, $p < 0.001$. Source data are provided as a Source Data file.

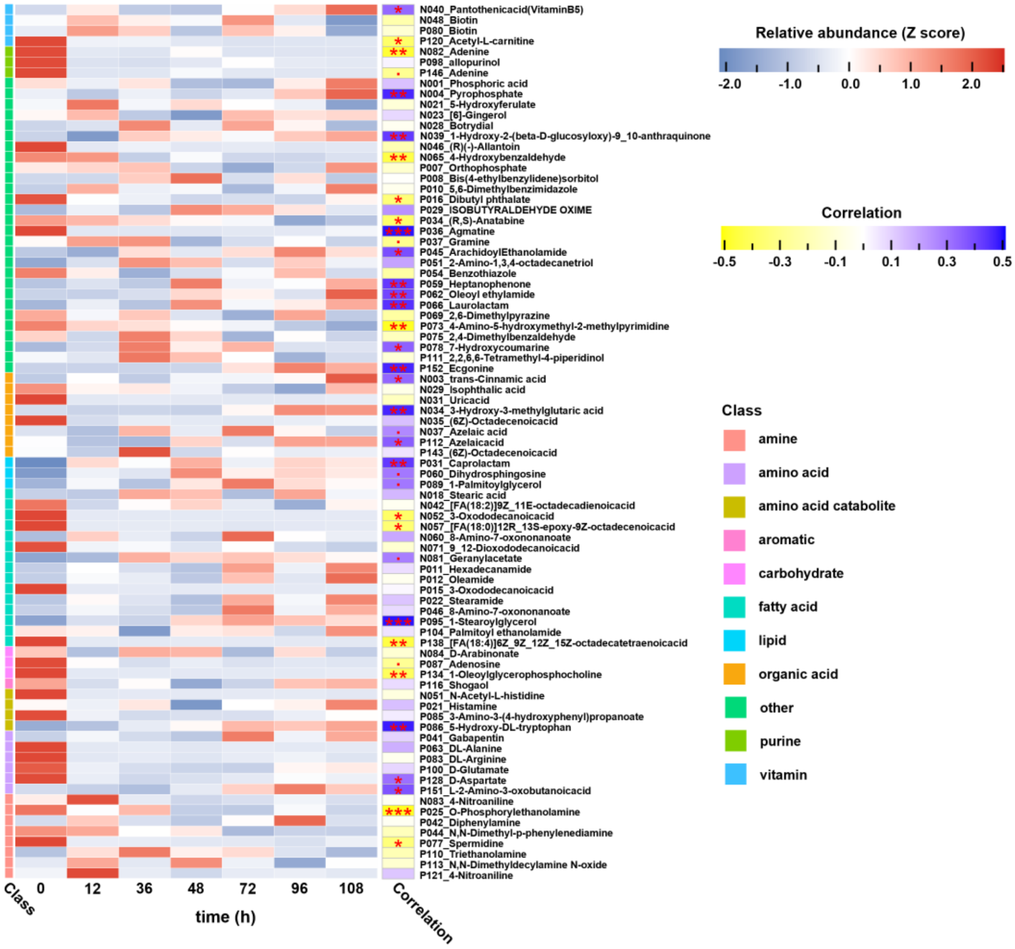

Supplementary Fig. 18. The dynamics of exometabolites in cluster 3 (n = 6 biological replicates for each time point). Spearman correlation coefficients between metabolite abundance and the incubation period were determined. *, $p < 0.05$; **, $p < 0.01$; ***, $p < 0.001$. Source data are provided as a Source Data file.

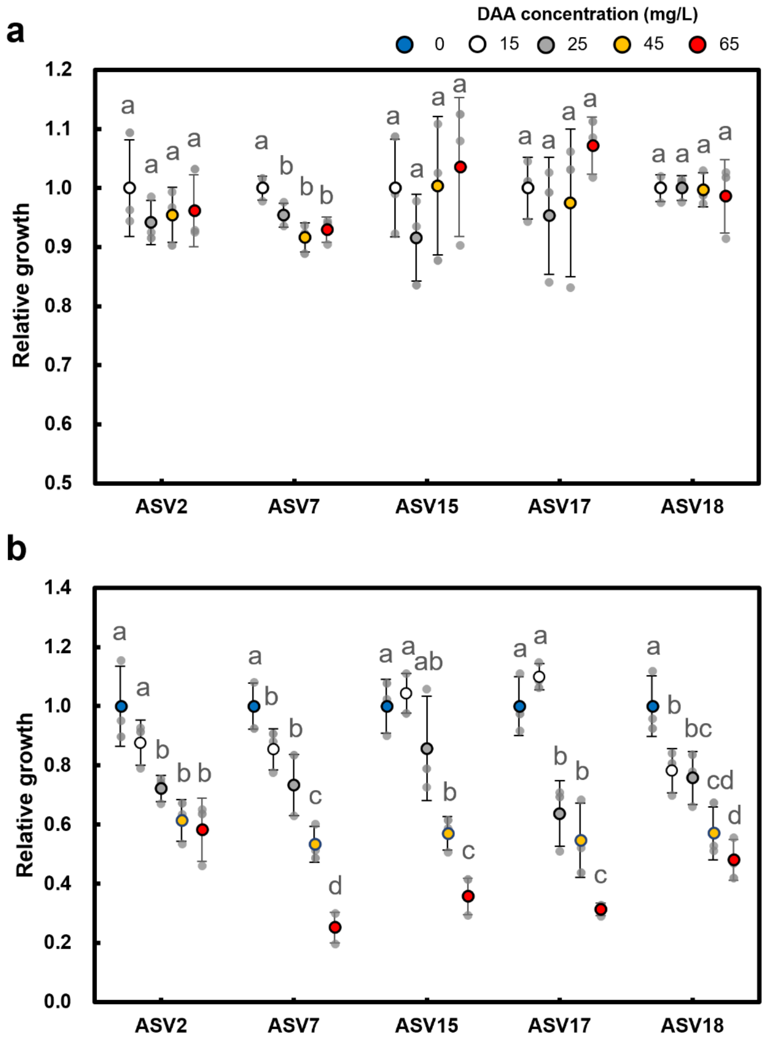

Supplementary Fig. 19. The relative growth of *Arthrobacter* strains in ISEM (a) and M9 minimal medium (b) supplemented with different levels of DAA after 48 h. A concentrated DAA stock solution with equal amounts of six DAAs (D-Val, D-Met, D-Leu, D-Phe, D-Thr and D-Trp) was diluted in ISEM and M9 medium to prepare different working concentrations. The growth (OD_{600}) in ISEM and M9 medium were normalized to those at the lowest DAA concentrations. Three independent replicates were carried out. Data are presented as mean values ± standard deviation. Different letters indicate significant differences in the relative growth of individual strains at different DAA levels ($p < 0.05$, one-way ANOVA), whilst shared letters indicate no statistical difference. For exact p values, see Supplementary Table 7.

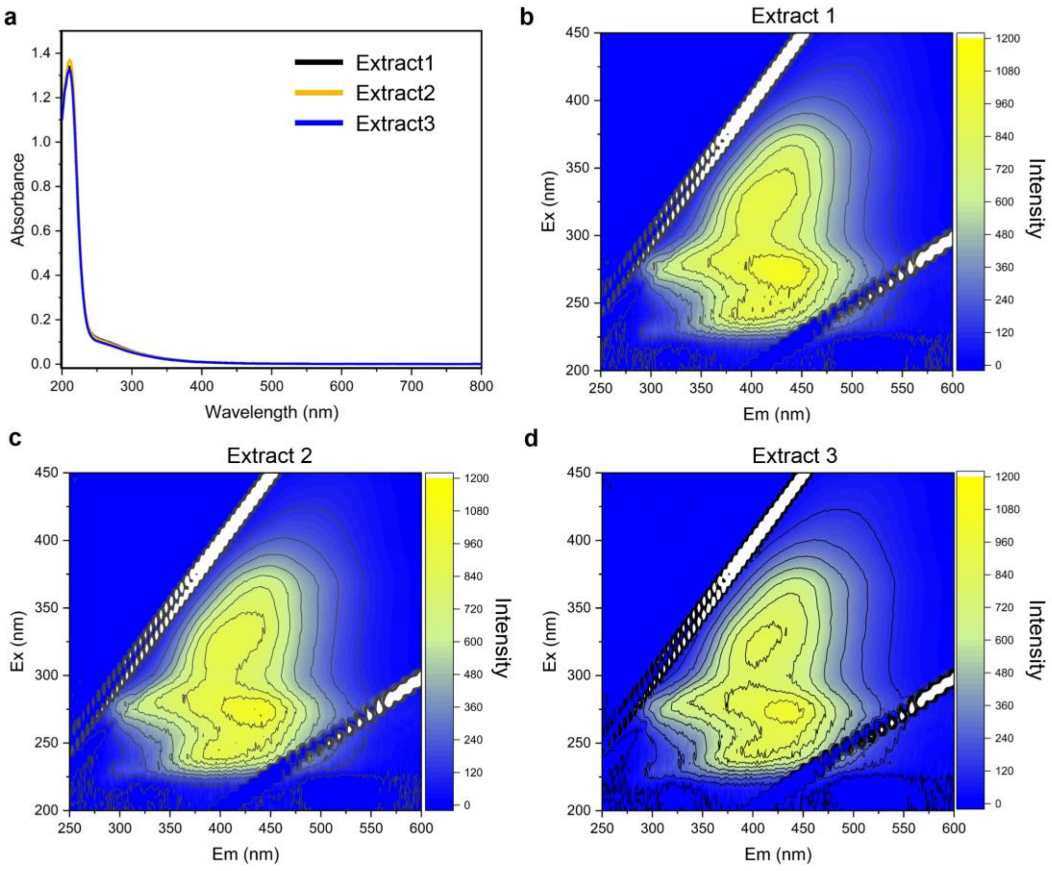

Supplementary Fig. 20. UV–vis absorption (a) and 3D-EEM fluorescence (b-d) spectra of ISEM from three independent batches. Similar spectroscopic features indicated a similar chemical composition of soil extract media.

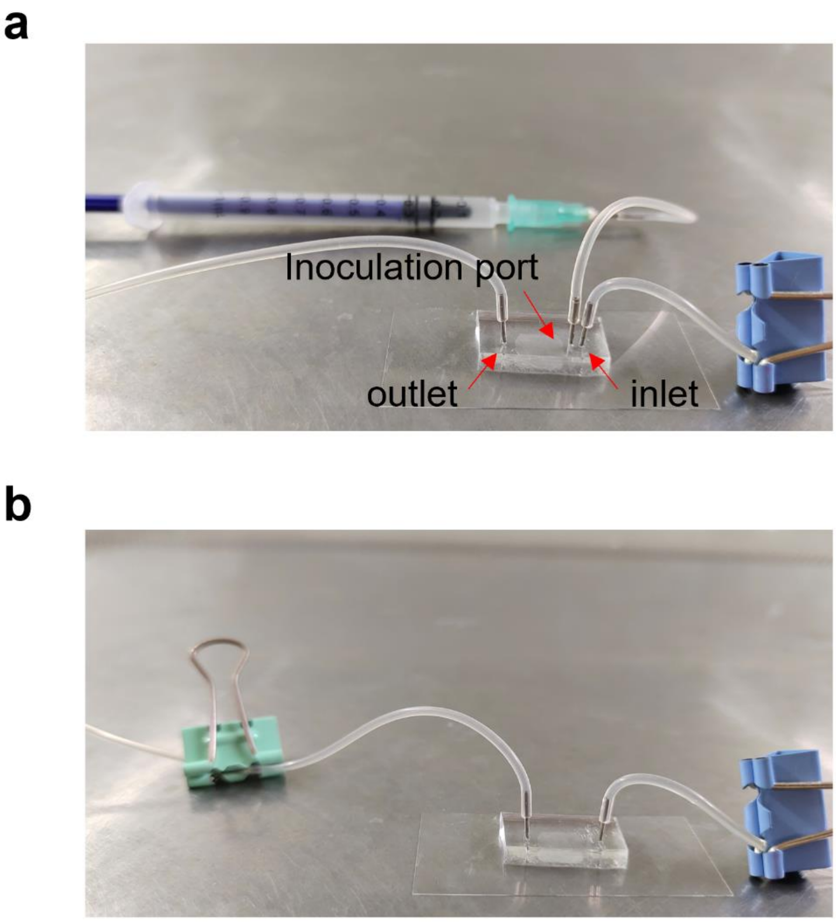

Supplementary Fig. 21. The inoculation of the microfluidic device. Bacterial suspension was introduced into the microfluidic chamber through an inoculation port located downstream of the medium inlet (a). The inlet and outlet tubing were clamped for one hour after inoculation to allow initial attachment (b).

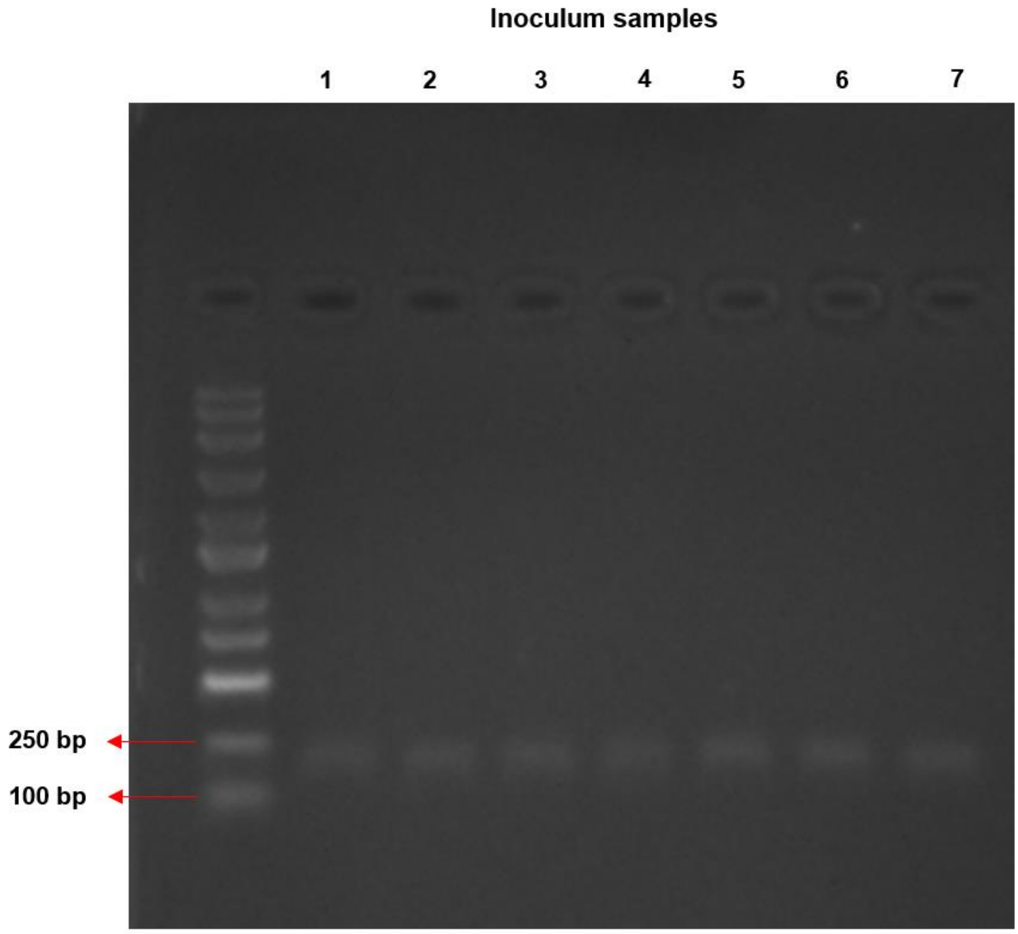

Supplementary Fig. 22. Gel electrophoresis of qPCR products amplified using the primer pair Eub338F/Eub518R. Lanes 1-7 are products derived from the inoculum samples. The PCR and gel electrophoresis were repeated three times and consistent results were obtained.

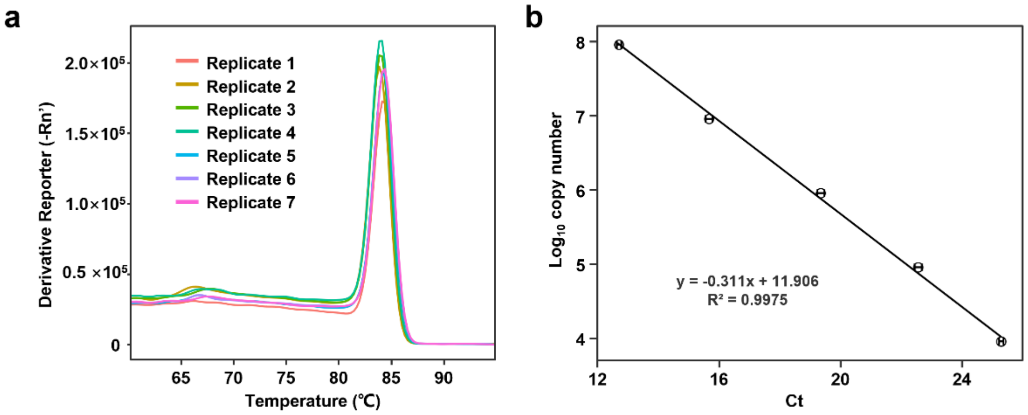

Supplementary Fig. 23. Melting curve analysis of qPCR products obtained from the inoculum sample (a). 7 replicates were performed. The standard curve developed using serial dilutions of a plasmid containing the target sequence (b). The open circles indicate the average Ct value for three replicates and error bars represent standard deviations.

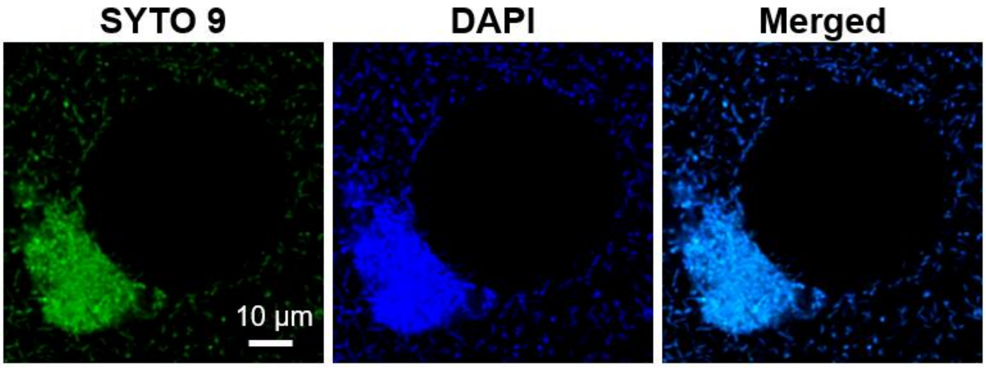

Supplementary Fig. 24. Representative CLSM images of biofilms stained with SYTO 9 and DAPI. The Pearson's correlation coefficient (PCC) was computed by ImageJ to evaluate the colocalization of these two fluorescent signals. The value of PCC is 0.977±0.005 (n = 6), indicating a high degree of colocalization.

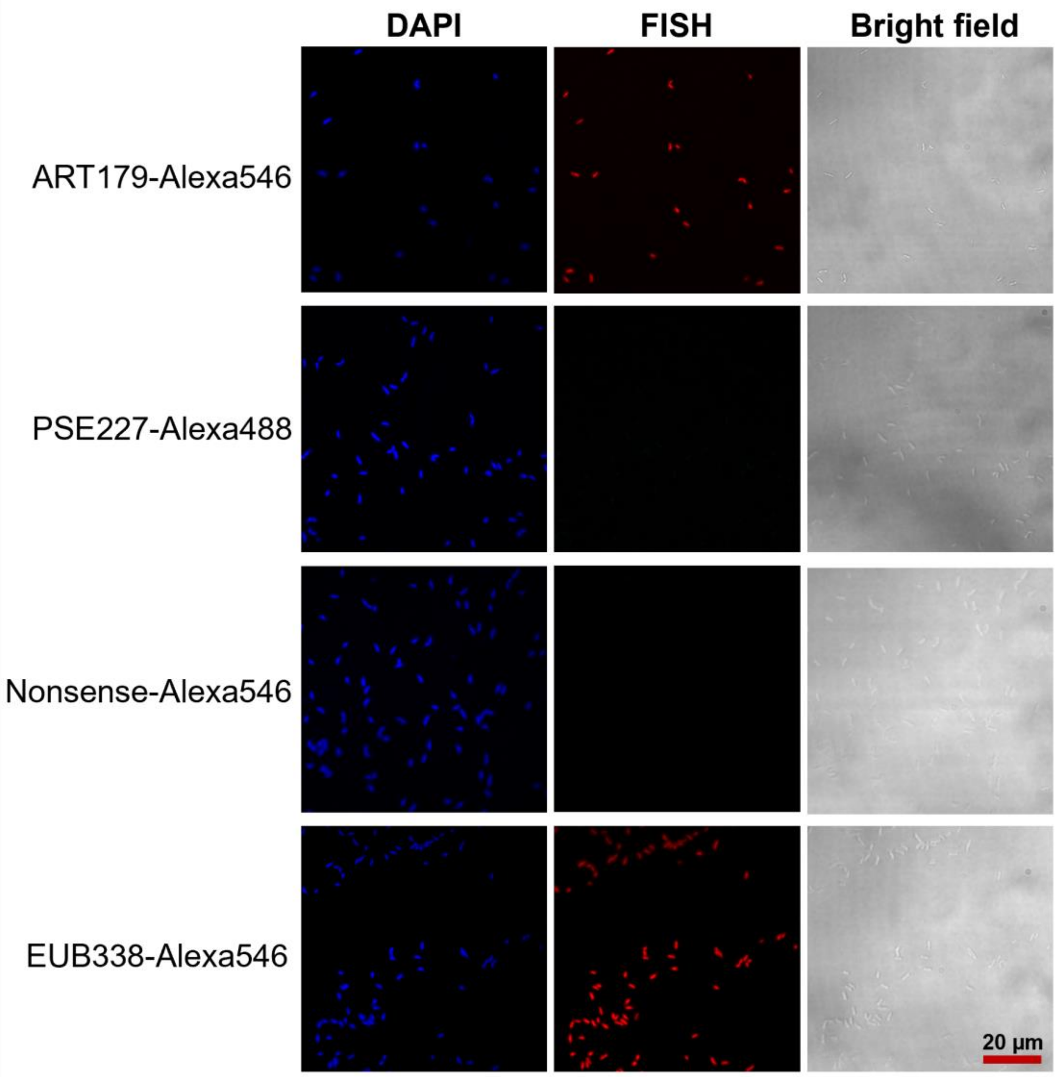

Supplementary Fig. 25. Fluorescence microscopy of ASV2 *A. ramosus* cells hybridized with different FISH probes. All the cells were stained with DAPI (blue fluorescence). ART179 and PSE227 were specific for *Arthrobacter* and *Pseudomonas*, respectively. Nonsense probe was used to exclude nonspecific binding. EUB338 identified all the bacterial cells was used as a positive control. Each treatment was repeated six times independently.

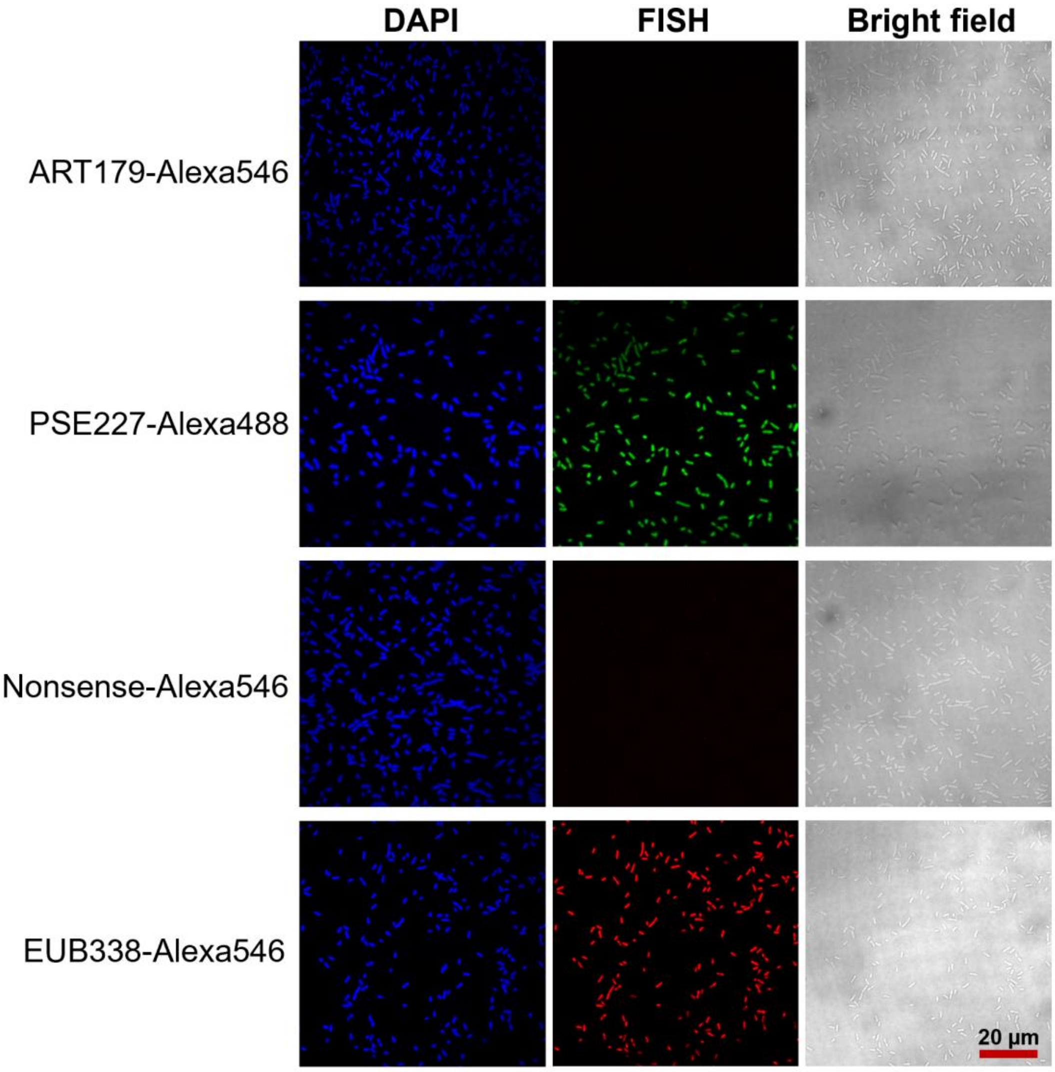

Supplementary Fig. 26. Fluorescence microscopy of ASV1 *P. fluorescens* cells hybridized with different FISH probes. All the cells were stained with DAPI (blue fluorescence). ART179 and PSE227 were specific for *Arthrobacter* and *Pseudomonas*, respectively. Nonsense probe was used to exclude nonspecific binding. EUB338 identified all the bacterial cells was used as a positive control. Each treatment was repeated six times independently.

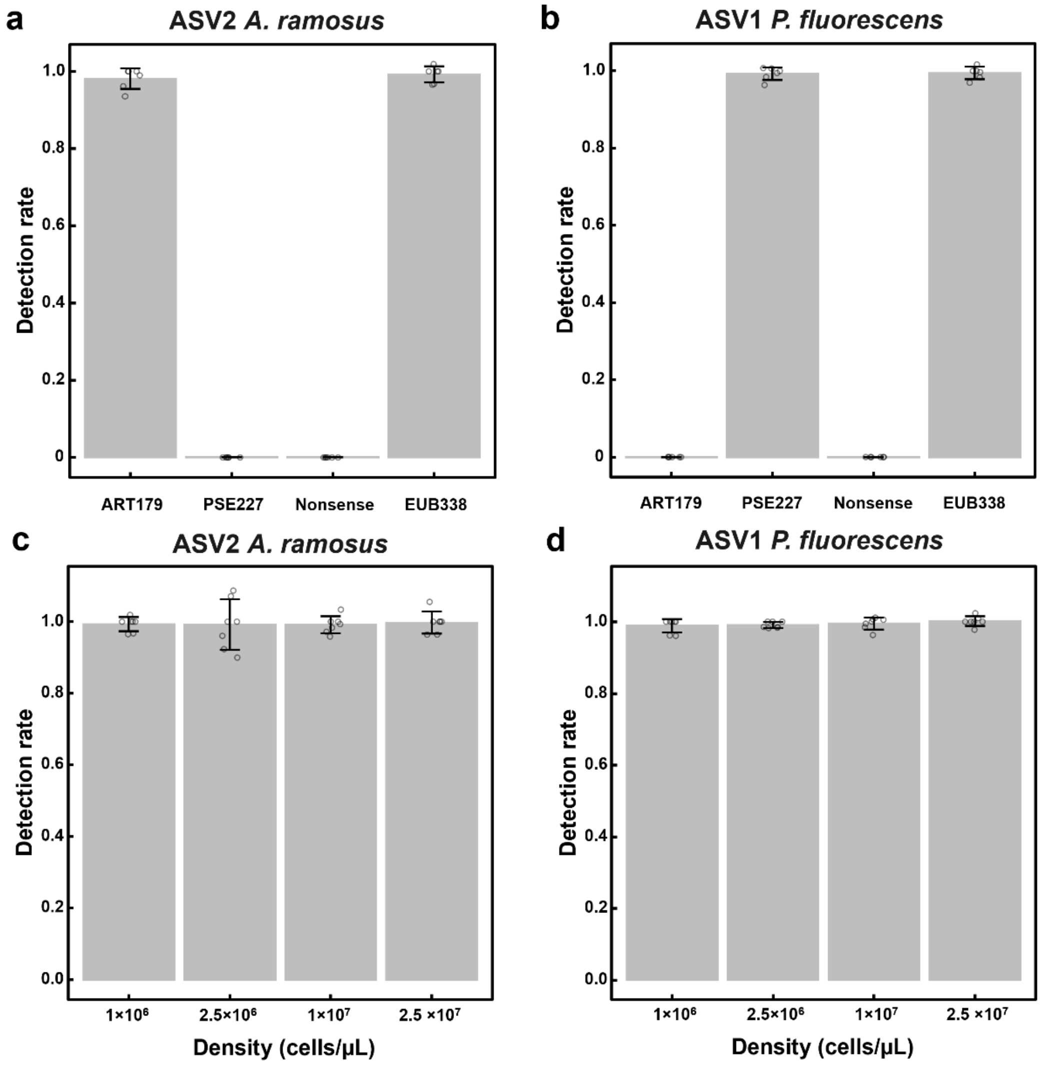

Supplementary Fig. 27. The detection rates of *Arthrobacter* and *Pseudomonas* cells with different FISH probes (a & b). Six independent replicates were performed for each treatment. Data are presented as mean values ± standard deviation. The detection rate is the proportion of DAPI-stained cells hybridized with fluorescent probes. The average detection rates for genus-specific and universal probes were higher than 98%, and no unspecific binding was observed. The bacterial suspensions were subjected to FISH analysis at different cell densities (c & d). Seven independent replicates were conducted for each density. The high average detection rates were maintained across a range of cell densities from 1.0×10^6 to 2.5×10^7 cells/μL.

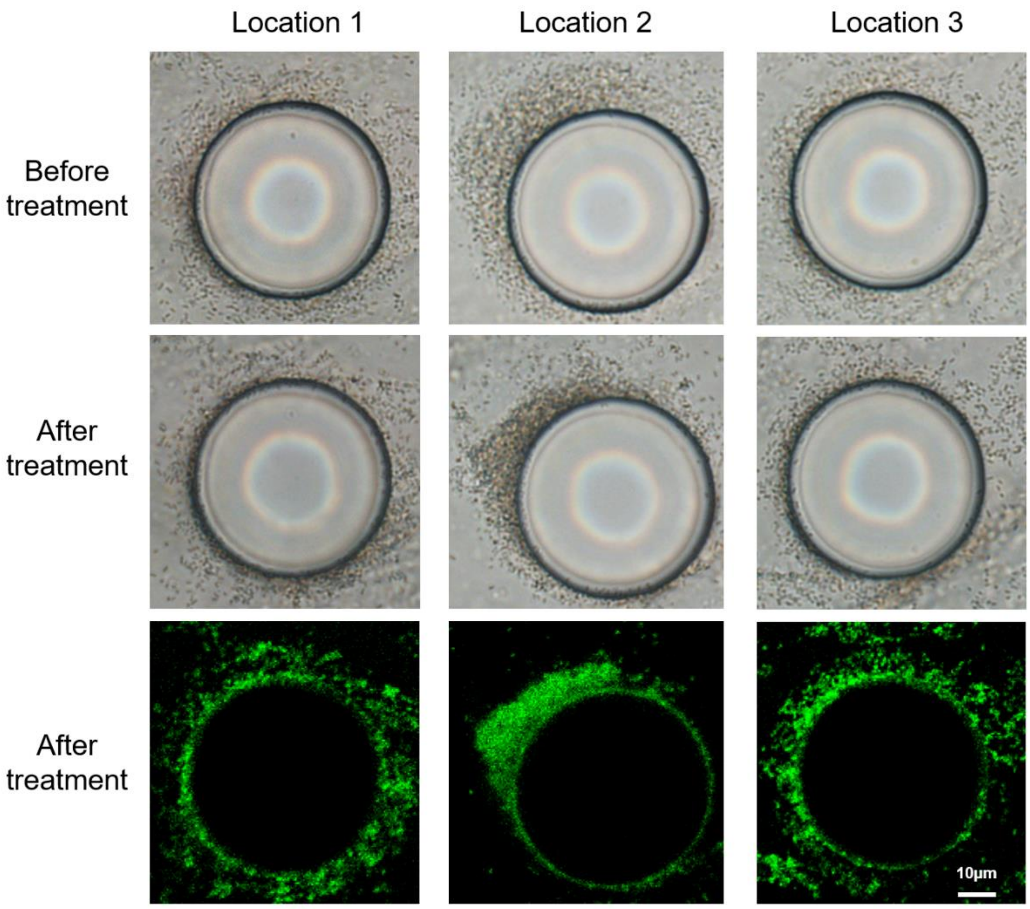

Supplementary Fig. 28. The biofilm morphologies at three different locations before and after the pretreatment for FISH analysis. The experiment was repeated in three independent microfluidic chips with similar results.

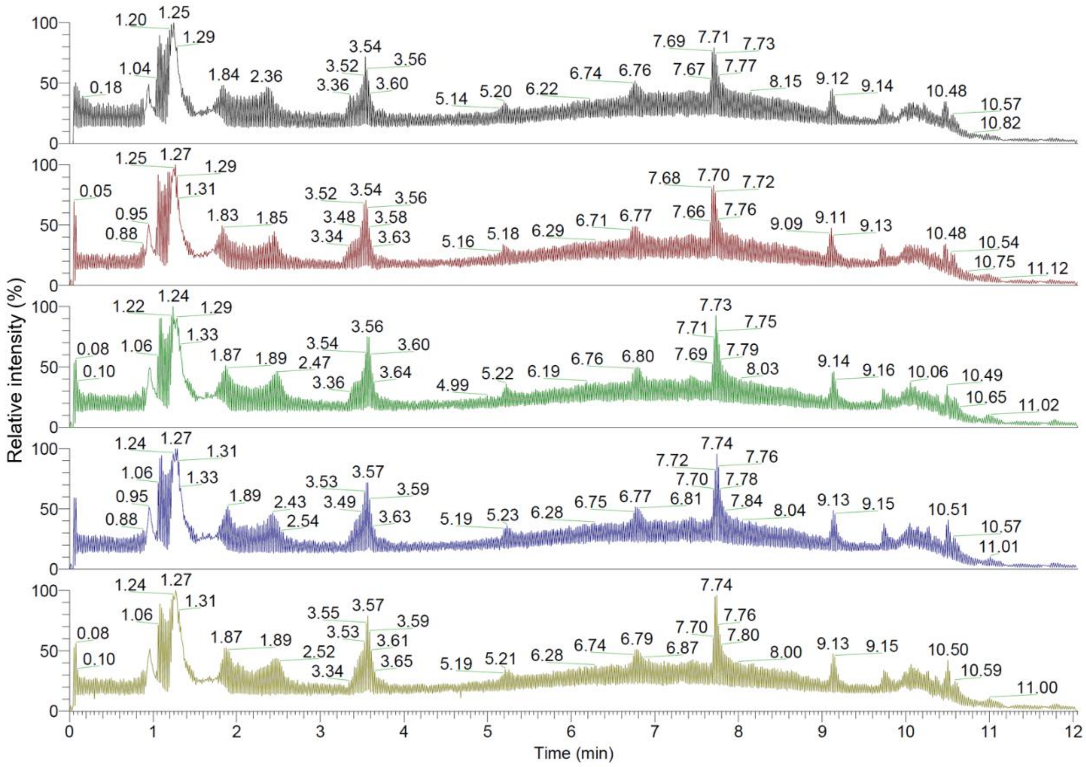

Supplementary Fig. 29. Total ion chromatograms of five pooled QC samples. The similar peak distributions indicated good system stability and reproducibility.

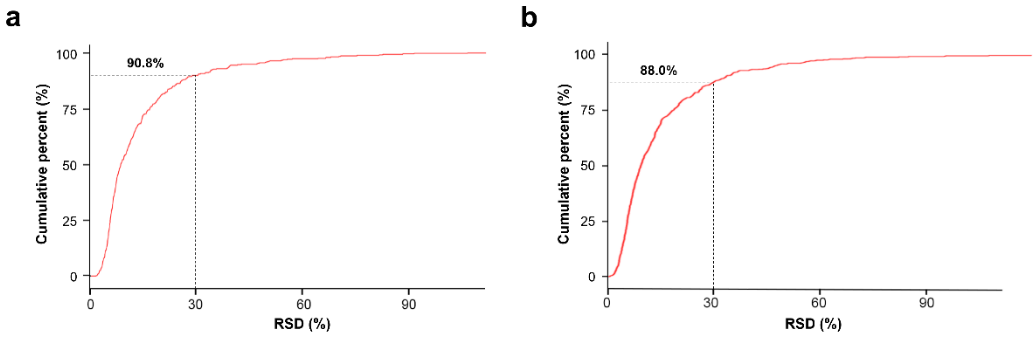

Supplementary Fig. 30. The cumulative relative standard deviation (RSD) distributions of ion peaks of QC samples detected in negative (a) and positive (b) modes. More than 85% of peaks had an RSD less than 30%, suggesting good stability and repeatability for further metabolomic investigations.

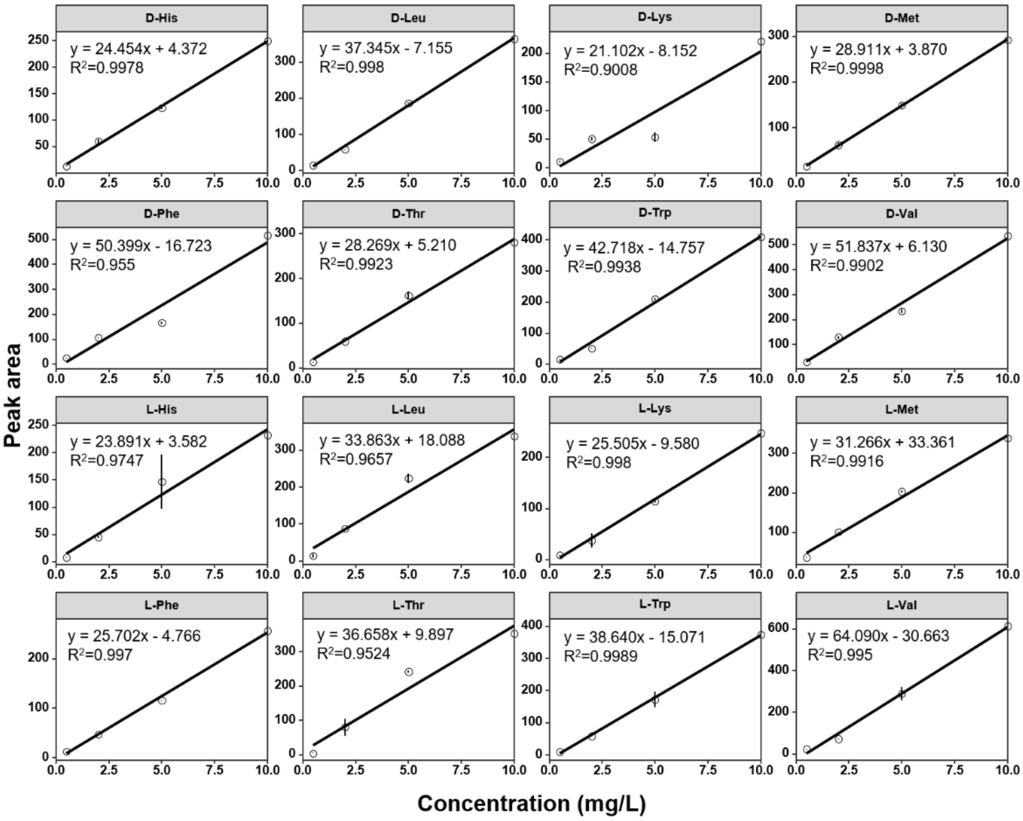

Supplementary Fig. 31. Standard curves of AAs determined by HPLC. For all AAs analyzed, good linearity was observed over the concentration range of 0.5-10 mg/L. The open circles indicate the average area of three replicates and error bars represent standard deviations.

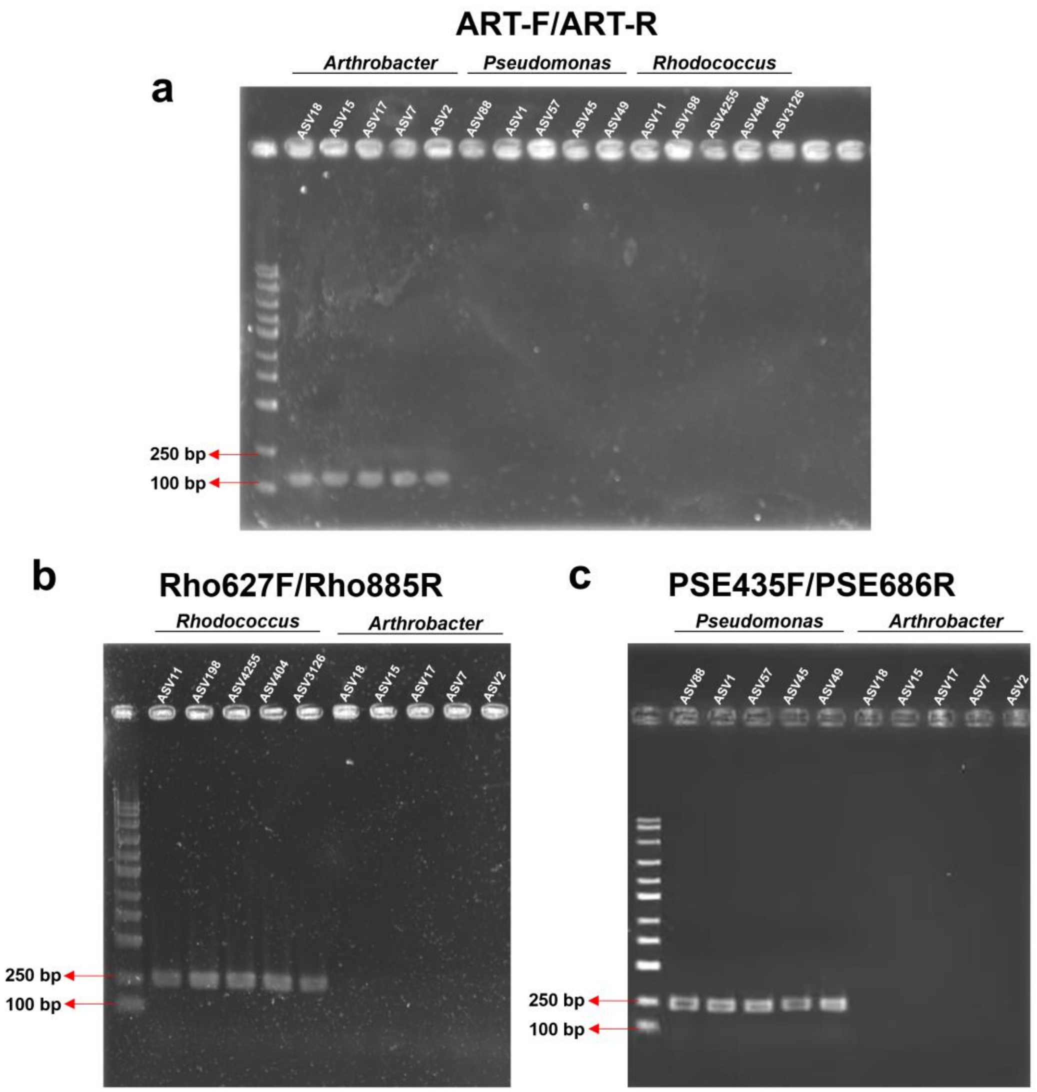

Supplementary Fig. 32. Gel electrophoresis of qPCR products amplified using the primer sets ART-F/ART-R (a), Rho627F/Rho885R (b) and PSE435F/PSE686R (c). Visible bands with appropriate size were only observed for target species, confirming the specificity of each primer sets. The PCR and gel electrophoresis were repeated three times and consistent results were obtained.

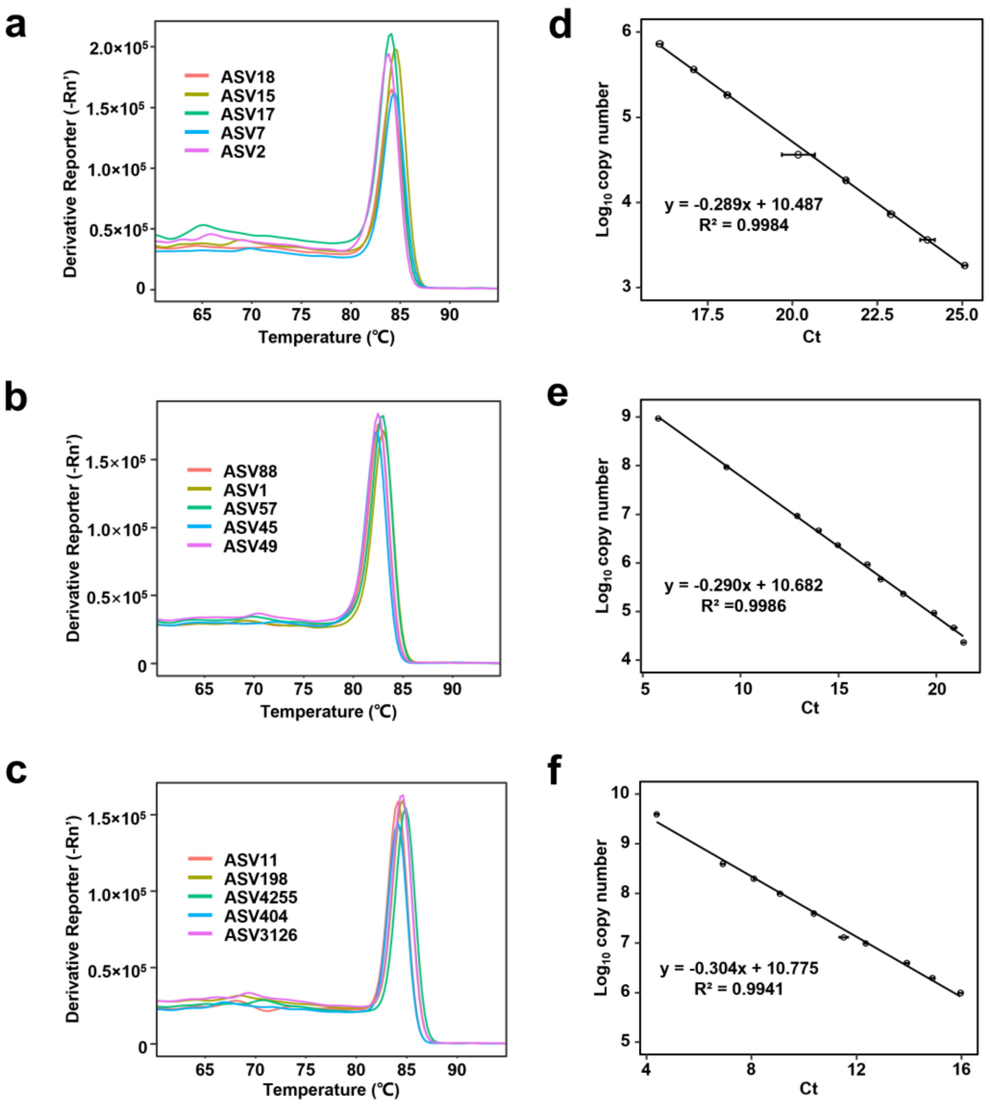

Supplementary Fig. 33. Melting curve analysis of qPCR products obtained from *Arthrobacter* (a), *Pseudomonas* (b) and *Rhodococcus* (c). The standard curves developed using serial dilutions of plasmids containing the target sequences from ASV2 *A. ramosus* (d), ASV1 *P. fluorescens* (e) and ASV11 *R. erythropolis* (f). The open circles indicate the average Ct value for three replicates, and error bars represent standard deviations.

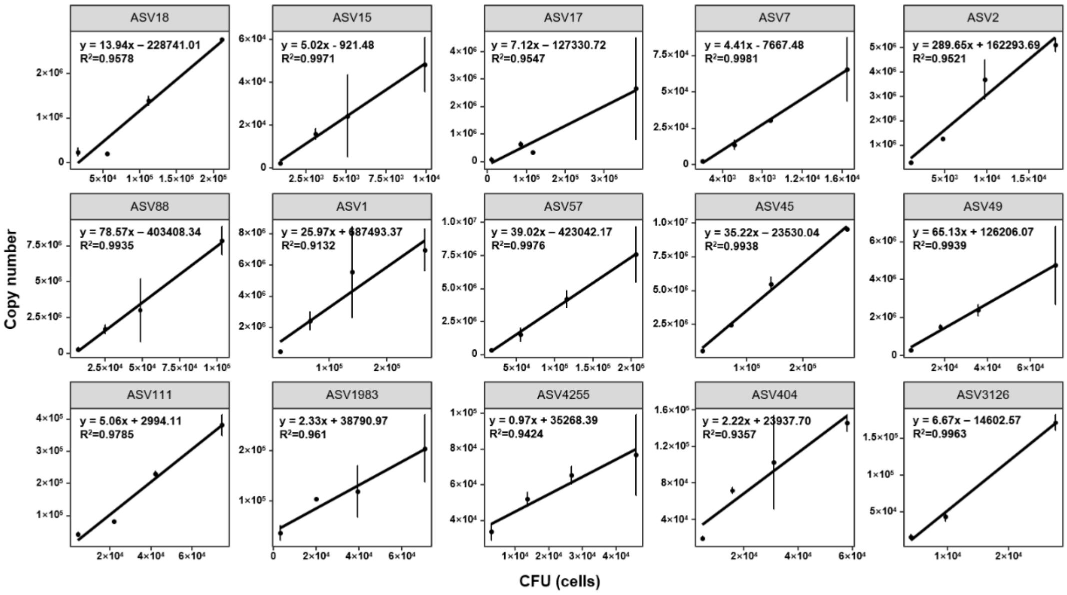

Supplementary Fig. 34. Linear correlations between the copy numbers determined by qPCR and cell numbers (CFU). The filled circles indicate the average area of three replicates and error bars represent standard deviations.

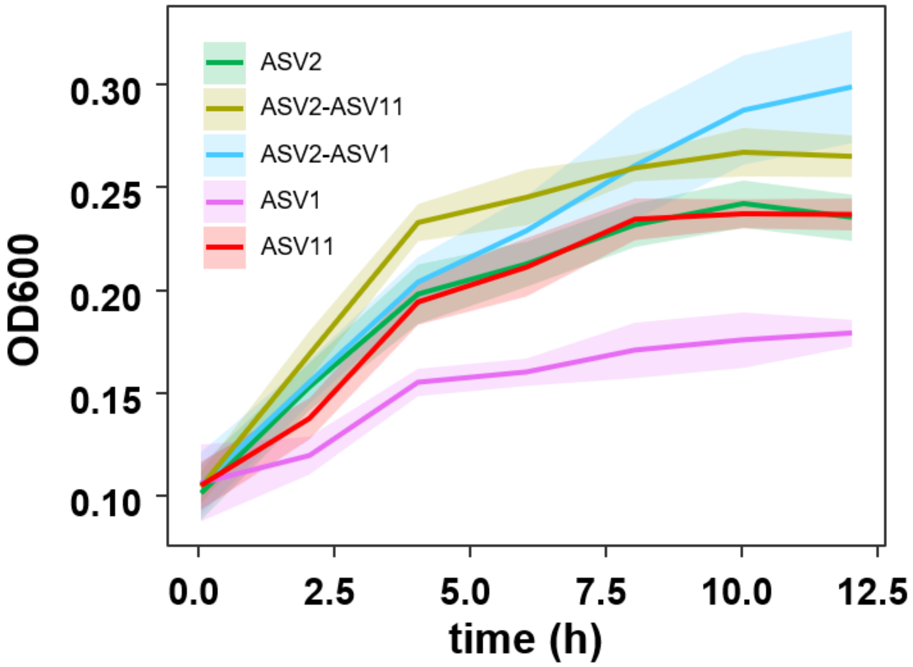

Supplementary Fig. 35. The growth of ASV2 *A. ramosus*, ASV1 *P. fluorescens* and ASV11 *R. erythropolis* in monoculture and co-culture in ISEM. The shaded areas represent the standard deviation calculated from three biological replicates.

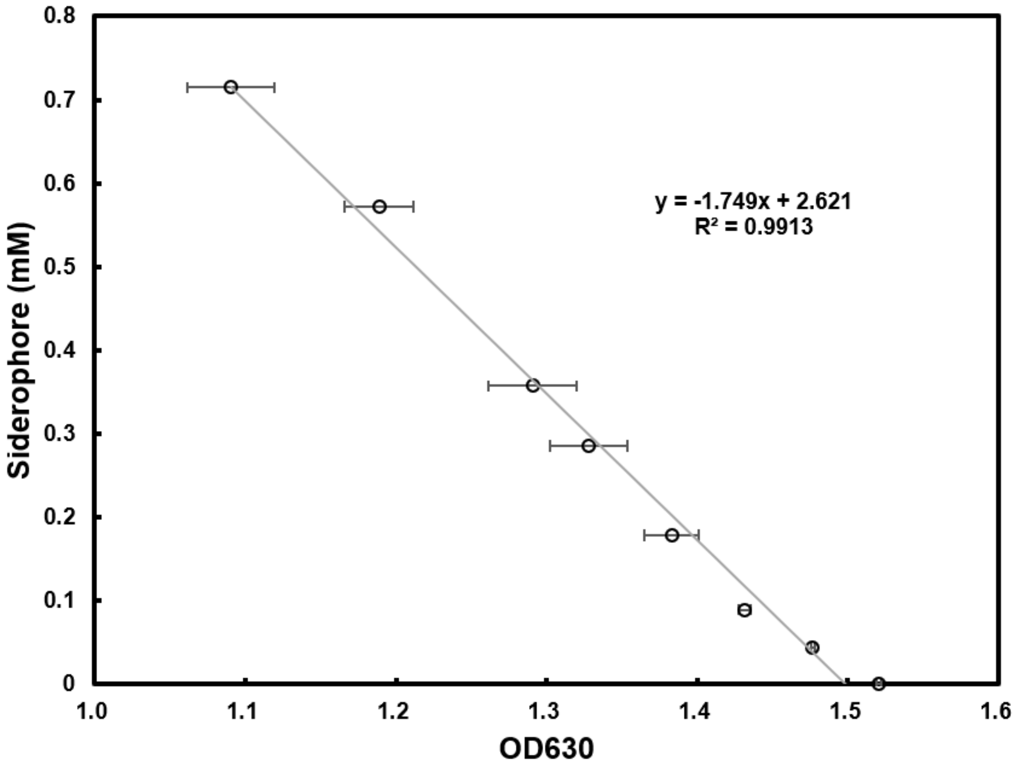

Supplementary Fig. 36. The standard curve for siderophore measurement constructed using Enterobactin. The open symbols indicate the average OD630 for three replicates at the same siderophore concentration, and error bars represent standard deviations of the measurement. The grey line represents the fitted standard curve siderophore (mM) = -1.749 × OD630 + 2.621.

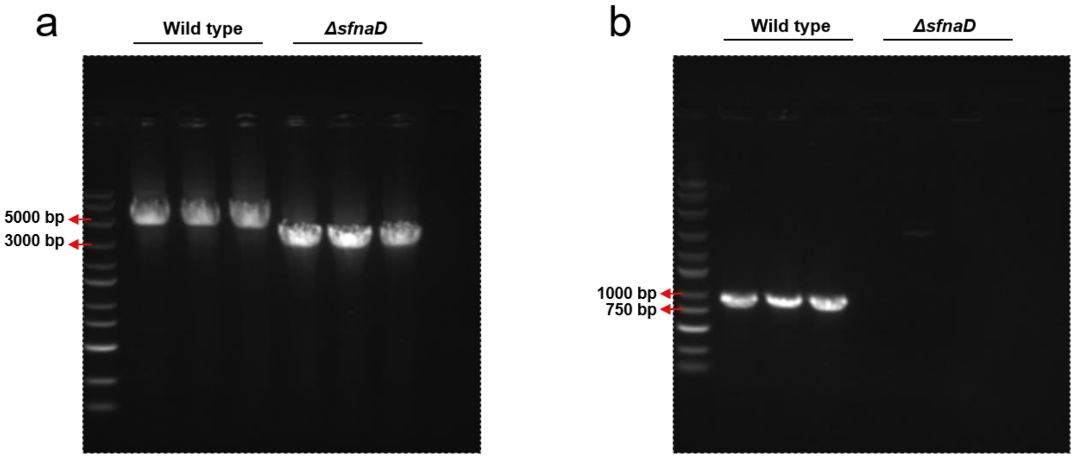

Supplementary Fig. 37. The deletion of *sfnaD* verified by PCR amplification and gel electrophoresis. The PCR product amplified from Δ*sfnaD* using the outside primers Out-sfnaD-S/Out-sfnaD-A is 1,444 bp smaller than that from the wild-type (a). A 928-bp fragment was amplified from the wild-type using the inside primers Ins-sfnaD-S/Ins-sfnaD-A, while no product was obtained in Δ*sfnaD* (b). The PCR and gel electrophoresis were repeated three times and consistent results were obtained.

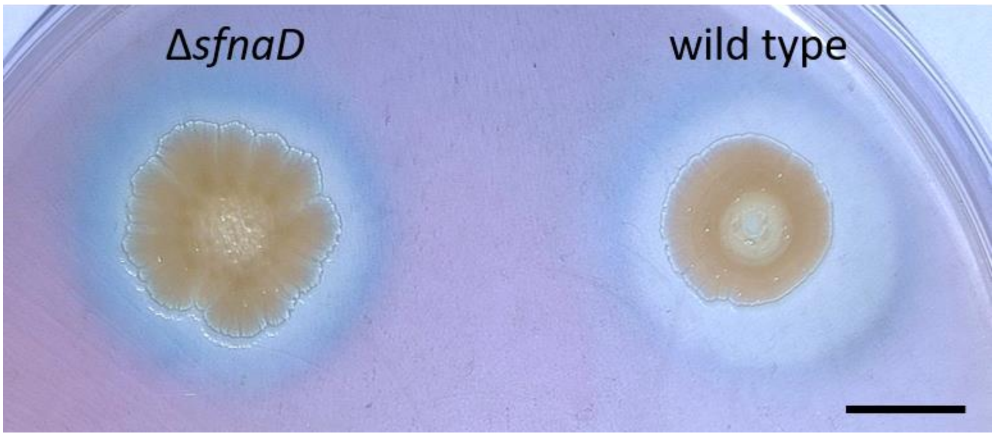

Supplementary Fig. 38. Colonies of Δ*sfnaD* and wild type on the ISEM agar plate containing 10% CAS assay solution. The Δ*sfnaD* colony with a reduced halo size indicated lower siderophore production compared to that of the wild type. Scale bar represents 1 cm.

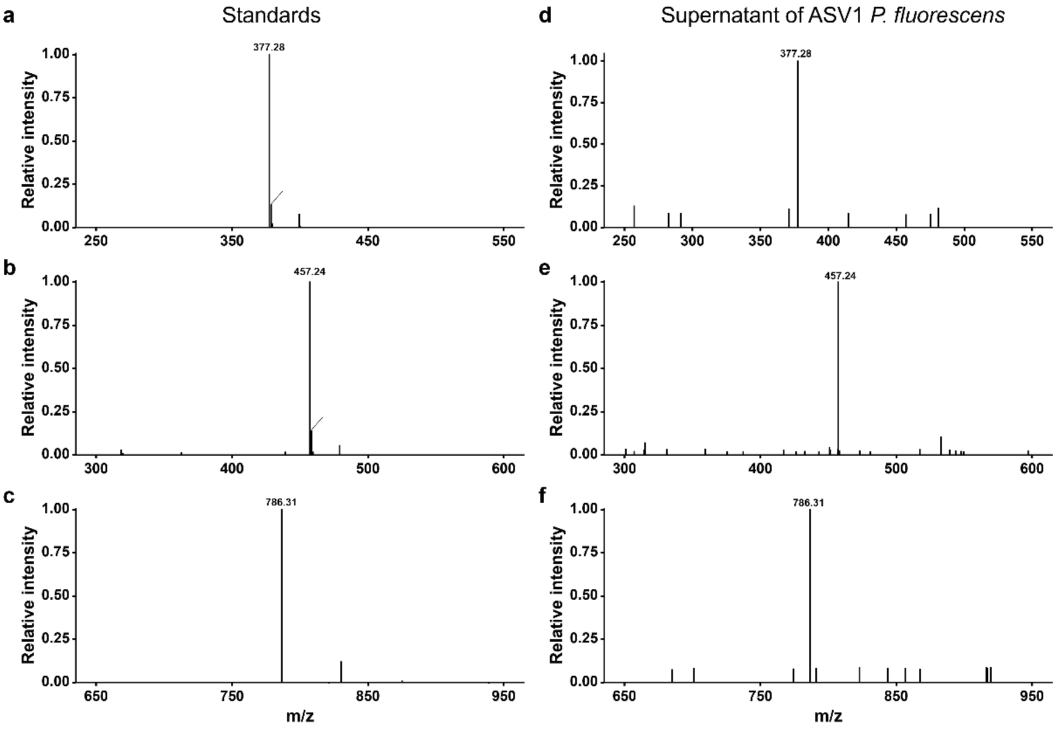

Supplementary Fig. 39. Mass spectra of RF (a), FMN (b), FAD (c) standards and the supernatant of ASV1 *P. fluorescens* cultivated in ISEM.

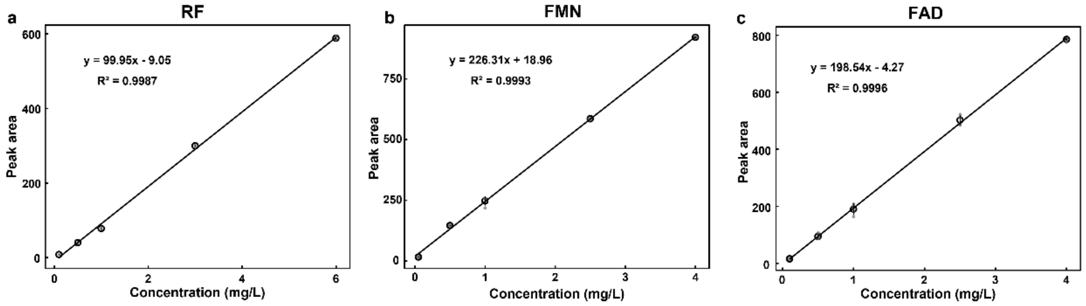

Supplementary Fig. 40. Calibration curves of RF, FMN and FAD determined by HPLC. The open circles indicate the average area of four replicates and error bars represent standard deviations.

Supplementary Table 1. Statistical results of comparisons shown in Figure 2c. The *p* value was calculated using one-way ANOVA.

Process	Group 1	Group 2	F value	*p* value
deterministic	12 h	36 h	5.279	0.035
deterministic	12 h	48 h	11.653	0.004
deterministic	12 h	72 h	12.471	0.003
deterministic	12 h	96 h	11.153	0.004
deterministic	12 h	108 h	10.539	0.005
deterministic	36 h	48 h	0.348	0.563
deterministic	36 h	72 h	0.795	0.386
deterministic	36 h	96 h	0.569	0.462
deterministic	36 h	108 h	0.402	0.535
deterministic	48 h	72 h	0.164	0.691
deterministic	48 h	96 h	0.061	0.808
deterministic	48 h	108 h	0.009	0.924
deterministic	72 h	96 h	0.019	0.891
deterministic	72 h	108 h	0.081	0.779
deterministic	96 h	108 h	0.020	0.889
stochastic	12 h	36 h	5.279	0.035
stochastic	12 h	48 h	11.653	0.004
stochastic	12 h	72 h	12.471	0.003
stochastic	12 h	96 h	11.153	0.004
stochastic	12 h	108 h	10.539	0.005
stochastic	36 h	48 h	0.348	0.563
stochastic	36 h	72 h	0.795	0.386
stochastic	36 h	96 h	0.569	0.462
stochastic	36 h	108 h	0.402	0.535
stochastic	48 h	72 h	0.164	0.691
stochastic	48 h	96 h	0.061	0.808
stochastic	48 h	108 h	0.009	0.924
stochastic	72 h	96 h	0.019	0.891
stochastic	72 h	108 h	0.081	0.779
stochastic	96 h	108 h	0.020	0.889

Supplementary Table 2. Statistical results of comparisons shown in Supplementary Figure 5. The p value was determined by two-tailed Student's t-test.

Combination	Group 1	Group 2	p value
ASV2-ASV1	Plankton-Mono-ASV2	Plankton-Coculture-ASV2	0.034
ASV2-ASV1	Plankton-Mono-ASV1	Plankton-Coculture-ASV1	0.613
ASV2-ASV1	Biofilm-Mono-ASV2	Biofilm -Coculture-ASV2	0.103
ASV2-ASV1	Biofilm -Mono-ASV1	Biofilm -Coculture-ASV1	2.051×10^{-4}
ASV2-ASV1	Total-Mono-ASV2	Total -Coculture-ASV2	0.029
ASV2-ASV1	Total -Mono-ASV1	Total -Coculture-ASV1	0.003
ASV2-ASV57	Plankton-Mono-ASV2	Plankton-Coculture-ASV2	0.011
ASV2-ASV57	Plankton-Mono-ASV57	Plankton-Coculture-ASV57	0.972
ASV2-ASV57	Biofilm-Mono-ASV2	Biofilm -Coculture-ASV2	0.658
ASV2-ASV57	Biofilm -Mono-ASV57	Biofilm -Coculture-ASV57	0.613
ASV2-ASV57	Total-Mono-ASV2	Total -Coculture-ASV2	0.016
ASV2-ASV57	Total -Mono-ASV57	Total -Coculture-ASV57	0.594
ASV2-ASV45	Plankton-Mono-ASV2	Plankton-Coculture-ASV2	0.017
ASV2-ASV45	Plankton-Mono-ASV45	Plankton-Coculture-ASV45	0.137
ASV2-ASV45	Biofilm-Mono-ASV2	Biofilm -Coculture-ASV2	0.038
ASV2-ASV45	Biofilm -Mono-ASV45	Biofilm -Coculture-ASV45	0.043
ASV2-ASV45	Total-Mono-ASV2	Total -Coculture-ASV2	0.006
ASV2-ASV45	Total -Mono-ASV45	Total -Coculture-ASV45	0.028
ASV2-ASV49	Plankton-Mono-ASV2	Plankton-Coculture-ASV2	0.035
ASV2-ASV49	Plankton-Mono-ASV49	Plankton-Coculture-ASV49	0.203
ASV2-ASV49	Biofilm-Mono-ASV2	Biofilm -Coculture-ASV2	0.031
ASV2-ASV49	Biofilm -Mono-ASV49	Biofilm -Coculture-ASV49	0.011
ASV2-ASV49	Total-Mono-ASV2	Total -Coculture-ASV2	0.004
ASV2-ASV49	Total -Mono-ASV49	Total -Coculture-ASV49	0.047
ASV2-ASV88	Plankton-Mono-ASV2	Plankton-Coculture-ASV2	0.021
ASV2-ASV88	Plankton-Mono-ASV88	Plankton-Coculture-ASV88	0.014
ASV2-ASV88	Biofilm-Mono-ASV2	Biofilm -Coculture-ASV2	0.046
ASV2-ASV88	Biofilm -Mono-ASV88	Biofilm -Coculture-ASV88	0.036
ASV2-ASV88	Total-Mono-ASV2	Total -Coculture-ASV2	0.010
ASV2-ASV88	Total -Mono-ASV88	Total -Coculture-ASV88	0.031
ASV7-ASV1	Plankton-Mono-ASV7	Plankton-Coculture-ASV7	0.018
ASV7-ASV1	Plankton-Mono-ASV1	Plankton-Coculture-ASV1	0.153
ASV7-ASV1	Biofilm-Mono-ASV7	Biofilm -Coculture-ASV7	0.068
ASV7-ASV1	Biofilm -Mono-ASV1	Biofilm -Coculture-ASV1	0.012
ASV7-ASV1	Total-Mono-ASV7	Total -Coculture-ASV7	0.018
ASV7-ASV1	Total -Mono-ASV1	Total -Coculture-ASV1	0.017
ASV7-ASV57	Plankton-Mono-ASV7	Plankton-Coculture-ASV7	0.051
ASV7-ASV57	Plankton-Mono-ASV57	Plankton-Coculture-ASV57	0.041
ASV7-ASV57	Biofilm-Mono-ASV7	Biofilm -Coculture-ASV7	0.056

ASV7-ASV57	Biofilm -Mono-ASV57	Biofilm -Coculture-ASV57	0.043
ASV7-ASV57	Total-Mono-ASV7	Total -Coculture-ASV7	0.022
ASV7-ASV57	Total -Mono-ASV57	Total -Coculture-ASV57	0.032
ASV7-ASV45	Plankton-Mono-ASV7	Plankton-Coculture-ASV7	0.044
ASV7-ASV45	Plankton-Mono-ASV45	Plankton-Coculture-ASV45	0.071
ASV7-ASV45	Biofilm-Mono-ASV7	Biofilm -Coculture-ASV7	0.014
ASV7-ASV45	Biofilm -Mono-ASV45	Biofilm -Coculture-ASV45	0.013
ASV7-ASV45	Total-Mono-ASV7	Total -Coculture-ASV7	0.036
ASV7-ASV45	Total -Mono-ASV45	Total -Coculture-ASV45	0.005
ASV7-ASV49	Plankton-Mono-ASV7	Plankton-Coculture-ASV7	0.027
ASV7-ASV49	Plankton-Mono-ASV49	Plankton-Coculture-ASV49	0.234
ASV7-ASV49	Biofilm-Mono-ASV7	Biofilm -Coculture-ASV7	0.114
ASV7-ASV49	Biofilm -Mono-ASV49	Biofilm -Coculture-ASV49	0.030
ASV7-ASV49	Total-Mono-ASV7	Total -Coculture-ASV7	0.032
ASV7-ASV49	Total -Mono-ASV49	Total -Coculture-ASV49	0.045
ASV7-ASV88	Plankton-Mono-ASV7	Plankton-Coculture-ASV7	0.015
ASV7-ASV88	Plankton-Mono-ASV88	Plankton-Coculture-ASV88	0.032
ASV7-ASV88	Biofilm-Mono-ASV7	Biofilm -Coculture-ASV7	0.056
ASV7-ASV88	Biofilm -Mono-ASV88	Biofilm -Coculture-ASV88	3.088×10^{-5}
ASV7-ASV88	Total-Mono-ASV7	Total -Coculture-ASV7	0.014
ASV7-ASV88	Total -Mono-ASV88	Total -Coculture-ASV88	2.750×10^{-4}
ASV15-ASV1	Plankton-Mono-ASV15	Plankton-Coculture-ASV15	0.217
ASV15-ASV1	Plankton-Mono-ASV1	Plankton-Coculture-ASV1	0.693
ASV15-ASV1	Biofilm-Mono-ASV15	Biofilm -Coculture-ASV15	0.058
ASV15-ASV1	Biofilm -Mono-ASV1	Biofilm -Coculture-ASV1	0.017
ASV15-ASV1	Total-Mono-ASV15	Total -Coculture-ASV15	0.102
ASV15-ASV1	Total -Mono-ASV1	Total -Coculture-ASV1	0.041
ASV15-ASV57	Plankton-Mono-ASV15	Plankton-Coculture-ASV15	0.110
ASV15-ASV57	Plankton-Mono-ASV57	Plankton-Coculture-ASV57	0.679
ASV15-ASV57	Biofilm-Mono-ASV15	Biofilm -Coculture-ASV15	0.001
ASV15-ASV57	Biofilm -Mono-ASV57	Biofilm -Coculture-ASV57	0.266
ASV15-ASV57	Total-Mono-ASV15	Total -Coculture-ASV15	0.027
ASV15-ASV57	Total -Mono-ASV57	Total -Coculture-ASV57	0.427
ASV15-ASV45	Plankton-Mono-ASV15	Plankton-Coculture-ASV15	0.029
ASV15-ASV45	Plankton-Mono-ASV45	Plankton-Coculture-ASV45	0.810
ASV15-ASV45	Biofilm-Mono-ASV15	Biofilm -Coculture-ASV15	0.369
ASV15-ASV45	Biofilm -Mono-ASV45	Biofilm -Coculture-ASV45	0.935
ASV15-ASV45	Total-Mono-ASV15	Total -Coculture-ASV15	0.022
ASV15-ASV45	Total -Mono-ASV45	Total -Coculture-ASV45	0.988
ASV15-ASV49	Plankton-Mono-ASV15	Plankton-Coculture-ASV15	0.099
ASV15-ASV49	Plankton-Mono-ASV49	Plankton-Coculture-ASV49	0.036
ASV15-ASV49	Biofilm-Mono-ASV15	Biofilm -Coculture-ASV15	0.459
ASV15-ASV49	Biofilm -Mono-ASV49	Biofilm -Coculture-ASV49	0.807
ASV15-ASV49	Total-Mono-ASV15	Total -Coculture-ASV15	0.145

ASV15-ASV49	Total -Mono-ASV49	Total -Coculture-ASV49	0.279
ASV15-ASV88	Plankton-Mono-ASV15	Plankton-Coculture-ASV15	0.007
ASV15-ASV88	Plankton-Mono-ASV88	Plankton-Coculture-ASV88	0.005
ASV15-ASV88	Biofilm-Mono-ASV15	Biofilm -Coculture-ASV15	0.157
ASV15-ASV88	Biofilm -Mono-ASV88	Biofilm -Coculture-ASV88	0.025
ASV15-ASV88	Total-Mono-ASV15	Total -Coculture-ASV15	0.005
ASV15-ASV88	Total -Mono-ASV88	Total -Coculture-ASV88	0.015
ASV17-ASV1	Plankton-Mono-ASV17	Plankton-Coculture-ASV17	0.004
ASV17-ASV1	Plankton-Mono-ASV1	Plankton-Coculture-ASV1	0.121
ASV17-ASV1	Biofilm-Mono-ASV17	Biofilm -Coculture-ASV17	0.036
ASV17-ASV1	Biofilm -Mono-ASV1	Biofilm -Coculture-ASV1	0.010
ASV17-ASV1	Total-Mono-ASV17	Total -Coculture-ASV17	0.003
ASV17-ASV1	Total -Mono-ASV1	Total -Coculture-ASV1	0.008
ASV17-ASV57	Plankton-Mono-ASV17	Plankton-Coculture-ASV17	0.013
ASV17-ASV57	Plankton-Mono-ASV57	Plankton-Coculture-ASV57	0.946
ASV17-ASV57	Biofilm-Mono-ASV17	Biofilm -Coculture-ASV17	0.183
ASV17-ASV57	Biofilm -Mono-ASV57	Biofilm -Coculture-ASV57	0.001
ASV17-ASV57	Total-Mono-ASV17	Total -Coculture-ASV17	0.015
ASV17-ASV57	Total -Mono-ASV57	Total -Coculture-ASV57	0.002
ASV17-ASV45	Plankton-Mono-ASV17	Plankton-Coculture-ASV17	0.029
ASV17-ASV45	Plankton-Mono-ASV45	Plankton-Coculture-ASV45	0.001
ASV17-ASV45	Biofilm-Mono-ASV17	Biofilm -Coculture-ASV17	0.066
ASV17-ASV45	Biofilm -Mono-ASV45	Biofilm -Coculture-ASV45	0.040
ASV17-ASV45	Total-Mono-ASV17	Total -Coculture-ASV17	0.015
ASV17-ASV45	Total -Mono-ASV45	Total -Coculture-ASV45	0.019
ASV17-ASV49	Plankton-Mono-ASV17	Plankton-Coculture-ASV17	0.012
ASV17-ASV49	Plankton-Mono-ASV49	Plankton-Coculture-ASV49	0.686
ASV17-ASV49	Biofilm-Mono-ASV17	Biofilm -Coculture-ASV17	0.360
ASV17-ASV49	Biofilm -Mono-ASV49	Biofilm -Coculture-ASV49	0.017
ASV17-ASV49	Total-Mono-ASV17	Total -Coculture-ASV17	0.024
ASV17-ASV49	Total -Mono-ASV49	Total -Coculture-ASV49	0.009
ASV17-ASV88	Plankton-Mono-ASV17	Plankton-Coculture-ASV17	0.004
ASV17-ASV88	Plankton-Mono-ASV88	Plankton-Coculture-ASV88	0.140
ASV17-ASV88	Biofilm-Mono-ASV17	Biofilm -Coculture-ASV17	0.135
ASV17-ASV88	Biofilm -Mono-ASV88	Biofilm -Coculture-ASV88	0.039
ASV17-ASV88	Total-Mono-ASV17	Total -Coculture-ASV17	0.031
ASV17-ASV88	Total -Mono-ASV88	Total -Coculture-ASV88	0.035
ASV18-ASV1	Plankton-Mono-ASV18	Plankton-Coculture-ASV18	0.006
ASV18-ASV1	Plankton-Mono-ASV1	Plankton-Coculture-ASV1	0.182
ASV18-ASV1	Biofilm-Mono-ASV18	Biofilm -Coculture-ASV18	0.566
ASV18-ASV1	Biofilm -Mono-ASV1	Biofilm -Coculture-ASV1	0.042
ASV18-ASV1	Total-Mono-ASV18	Total -Coculture-ASV18	0.037
ASV18-ASV1	Total -Mono-ASV1	Total -Coculture-ASV1	0.002
ASV18-ASV57	Plankton-Mono-ASV18	Plankton-Coculture-ASV18	0.944

ASV18-ASV57	Plankton-Mono-ASV57	Plankton-Coculture-ASV57	0.888
ASV18-ASV57	Biofilm-Mono-ASV18	Biofilm -Coculture-ASV18	0.124
ASV18-ASV57	Biofilm -Mono-ASV57	Biofilm -Coculture-ASV57	2.230×10^{-4}
ASV18-ASV57	Total-Mono-ASV18	Total -Coculture-ASV18	0.350
ASV18-ASV57	Total -Mono-ASV57	Total -Coculture-ASV57	2.999×10^{-4}
ASV18-ASV45	Plankton-Mono-ASV18	Plankton-Coculture-ASV18	0.012
ASV18-ASV45	Plankton-Mono-ASV45	Plankton-Coculture-ASV45	0.037
ASV18-ASV45	Biofilm-Mono-ASV18	Biofilm -Coculture-ASV18	0.199
ASV18-ASV45	Biofilm -Mono-ASV45	Biofilm -Coculture-ASV45	0.041
ASV18-ASV45	Total-Mono-ASV18	Total -Coculture-ASV18	0.011
ASV18-ASV45	Total -Mono-ASV45	Total -Coculture-ASV45	0.031
ASV18-ASV49	Plankton-Mono-ASV18	Plankton-Coculture-ASV18	0.005
ASV18-ASV49	Plankton-Mono-ASV49	Plankton-Coculture-ASV49	0.375
ASV18-ASV49	Biofilm-Mono-ASV18	Biofilm -Coculture-ASV18	0.485
ASV18-ASV49	Biofilm -Mono-ASV49	Biofilm -Coculture-ASV49	0.014
ASV18-ASV49	Total-Mono-ASV18	Total -Coculture-ASV18	0.010
ASV18-ASV49	Total -Mono-ASV49	Total -Coculture-ASV49	0.010
ASV18-ASV88	Plankton-Mono-ASV18	Plankton-Coculture-ASV18	0.035
ASV18-ASV88	Plankton-Mono-ASV88	Plankton-Coculture-ASV88	0.097
ASV18-ASV88	Biofilm-Mono-ASV18	Biofilm -Coculture-ASV18	0.898
ASV18-ASV88	Biofilm -Mono-ASV88	Biofilm -Coculture-ASV88	0.015
ASV18-ASV88	Total-Mono-ASV18	Total -Coculture-ASV18	0.036
ASV18-ASV88	Total -Mono-ASV88	Total -Coculture-ASV88	0.021

Supplementary Table 3. Statistical results of comparisons shown in Supplementary Figure 6. The p value was determined by two-tailed Student's t-test.

Combination	Group 1	Group 2	p value
ASV2-ASV11	Plankton-Mono-ASV2	Plankton-Coculture-ASV2	0.186
ASV2-ASV11	Plankton-Mono-ASV11	Plankton-Coculture-ASV11	0.012
ASV2-ASV11	Biofilm-Mono-ASV2	Biofilm -Coculture-ASV2	0.029
ASV2-ASV11	Biofilm -Mono-ASV11	Biofilm -Coculture-ASV11	0.018
ASV2-ASV11	Total-Mono-ASV2	Total -Coculture-ASV2	0.029
ASV2-ASV11	Total -Mono-ASV11	Total -Coculture-ASV11	0.052
ASV2-ASV198	Plankton-Mono-ASV2	Plankton-Coculture-ASV2	0.080
ASV2-ASV198	Plankton-Mono-ASV198	Plankton-Coculture-ASV198	0.455
ASV2-ASV198	Biofilm-Mono-ASV2	Biofilm -Coculture-ASV2	0.045
ASV2-ASV198	Biofilm -Mono-ASV198	Biofilm -Coculture-ASV198	0.922
ASV2-ASV198	Total-Mono-ASV2	Total -Coculture-ASV2	0.035
ASV2-ASV198	Total -Mono-ASV198	Total -Coculture-ASV198	0.607
ASV2-ASV404	Plankton-Mono-ASV2	Plankton-Coculture-ASV2	0.013
ASV2-ASV404	Plankton-Mono-ASV404	Plankton-Coculture-ASV404	0.103
ASV2-ASV404	Biofilm-Mono-ASV2	Biofilm -Coculture-ASV2	0.009
ASV2-ASV404	Biofilm -Mono-ASV404	Biofilm -Coculture-ASV404	0.029
ASV2-ASV404	Total-Mono-ASV2	Total -Coculture-ASV2	0.008
ASV2-ASV404	Total -Mono-ASV404	Total -Coculture-ASV404	0.042
ASV2-ASV3126	Plankton-Mono-ASV2	Plankton-Coculture-ASV2	0.022
ASV2-ASV3126	Plankton-Mono-ASV3126	Plankton-Coculture-ASV3126	0.352
ASV2-ASV3126	Biofilm-Mono-ASV2	Biofilm -Coculture-ASV2	0.184
ASV2-ASV3126	Biofilm -Mono-ASV3126	Biofilm -Coculture-ASV3126	0.045
ASV2-ASV3126	Total-Mono-ASV2	Total -Coculture-ASV2	0.040
ASV2-ASV3126	Total -Mono-ASV3126	Total -Coculture-ASV3126	0.028
ASV2-ASV4255	Plankton-Mono-ASV2	Plankton-Coculture-ASV2	0.059
ASV2-ASV4255	Plankton-Mono-ASV4255	Plankton-Coculture-ASV4255	0.795
ASV2-ASV4255	Biofilm-Mono-ASV2	Biofilm -Coculture-ASV2	0.016
ASV2-ASV4255	Biofilm -Mono-ASV4255	Biofilm -Coculture-ASV4255	0.007
ASV2-ASV4255	Total-Mono-ASV2	Total -Coculture-ASV2	0.014
ASV2-ASV4255	Total -Mono-ASV4255	Total -Coculture-ASV4255	0.024
ASV7-ASV11	Plankton-Mono-ASV7	Plankton-Coculture-ASV7	0.056
ASV7-ASV11	Plankton-Mono-ASV11	Plankton-Coculture-ASV11	0.139
ASV7-ASV11	Biofilm-Mono-ASV7	Biofilm -Coculture-ASV7	0.289
ASV7-ASV11	Biofilm -Mono-ASV11	Biofilm -Coculture-ASV11	0.340
ASV7-ASV11	Total-Mono-ASV7	Total -Coculture-ASV7	0.052
ASV7-ASV11	Total -Mono-ASV11	Total -Coculture-ASV11	0.024
ASV7-ASV198	Plankton-Mono-ASV7	Plankton-Coculture-ASV7	0.010
ASV7-ASV198	Plankton-Mono-ASV198	Plankton-Coculture-ASV198	0.560
ASV7-ASV198	Biofilm-Mono-ASV7	Biofilm -Coculture-ASV7	0.016

ASV7-ASV198	Biofilm -Mono-ASV198	Biofilm -Coculture-ASV198	0.510
ASV7-ASV198	Total-Mono-ASV7	Total -Coculture-ASV7	0.006
ASV7-ASV198	Total -Mono-ASV198	Total -Coculture-ASV198	0.943
ASV7-ASV404	Plankton-Mono-ASV7	Plankton-Coculture-ASV7	0.118
ASV7-ASV404	Plankton-Mono-ASV404	Plankton-Coculture-ASV404	0.160
ASV7-ASV404	Biofilm-Mono-ASV7	Biofilm -Coculture-ASV7	0.028
ASV7-ASV404	Biofilm -Mono-ASV404	Biofilm -Coculture-ASV404	0.009
ASV7-ASV404	Total-Mono-ASV7	Total -Coculture-ASV7	0.028
ASV7-ASV404	Total -Mono-ASV404	Total -Coculture-ASV404	0.008
ASV7-ASV3126	Plankton-Mono-ASV7	Plankton-Coculture-ASV7	0.149
ASV7-ASV3126	Plankton-Mono-ASV3126	Plankton-Coculture-ASV3126	0.014
ASV7-ASV3126	Biofilm-Mono-ASV7	Biofilm -Coculture-ASV7	0.114
ASV7-ASV3126	Biofilm -Mono-ASV3126	Biofilm -Coculture-ASV3126	0.043
ASV7-ASV3126	Total-Mono-ASV7	Total -Coculture-ASV7	0.139
ASV7-ASV3126	Total -Mono-ASV3126	Total -Coculture-ASV3126	0.028
ASV7-ASV4255	Plankton-Mono-ASV7	Plankton-Coculture-ASV7	0.042
ASV7-ASV4255	Plankton-Mono-ASV4255	Plankton-Coculture-ASV4255	5.589×10^{-5}
ASV7-ASV4255	Biofilm-Mono-ASV7	Biofilm -Coculture-ASV7	0.041
ASV7-ASV4255	Biofilm -Mono-ASV4255	Biofilm -Coculture-ASV4255	0.043
ASV7-ASV4255	Total-Mono-ASV7	Total -Coculture-ASV7	0.036
ASV7-ASV4255	Total -Mono-ASV4255	Total -Coculture-ASV4255	0.013
ASV15-ASV11	Plankton-Mono-ASV15	Plankton-Coculture-ASV15	0.027
ASV15-ASV11	Plankton-Mono-ASV11	Plankton-Coculture-ASV11	0.379
ASV15-ASV11	Biofilm-Mono-ASV15	Biofilm -Coculture-ASV15	0.536
ASV15-ASV11	Biofilm -Mono-ASV11	Biofilm -Coculture-ASV11	0.042
ASV15-ASV11	Total-Mono-ASV15	Total -Coculture-ASV15	0.021
ASV15-ASV11	Total -Mono-ASV11	Total -Coculture-ASV11	0.039
ASV15-ASV198	Plankton-Mono-ASV15	Plankton-Coculture-ASV15	0.143
ASV15-ASV198	Plankton-Mono-ASV198	Plankton-Coculture-ASV198	0.321
ASV15-ASV198	Biofilm-Mono-ASV15	Biofilm -Coculture-ASV15	0.030
ASV15-ASV198	Biofilm -Mono-ASV198	Biofilm -Coculture-ASV198	0.755
ASV15-ASV198	Total-Mono-ASV15	Total -Coculture-ASV15	0.048
ASV15-ASV198	Total -Mono-ASV198	Total -Coculture-ASV198	0.804
ASV15-ASV404	Plankton-Mono-ASV15	Plankton-Coculture-ASV15	0.115
ASV15-ASV404	Plankton-Mono-ASV404	Plankton-Coculture-ASV404	0.973
ASV15-ASV404	Biofilm-Mono-ASV15	Biofilm -Coculture-ASV15	0.257
ASV15-ASV404	Biofilm -Mono-ASV404	Biofilm -Coculture-ASV404	0.038
ASV15-ASV404	Total-Mono-ASV15	Total -Coculture-ASV15	0.044
ASV15-ASV404	Total -Mono-ASV404	Total -Coculture-ASV404	0.025
ASV15-ASV3126	Plankton-Mono-ASV15	Plankton-Coculture-ASV15	0.062
ASV15-ASV3126	Plankton-Mono-ASV3126	Plankton-Coculture-ASV3126	0.620
ASV15-ASV3126	Biofilm-Mono-ASV15	Biofilm -Coculture-ASV15	0.252
ASV15-ASV3126	Biofilm -Mono-ASV3126	Biofilm -Coculture-ASV3126	0.001
ASV15-ASV3126	Total-Mono-ASV15	Total -Coculture-ASV15	0.021

ASV15-ASV3126	Total -Mono-ASV3126	Total -Coculture-ASV3126	3.649×10^{-4}
ASV15-ASV4255	Plankton-Mono-ASV15	Plankton-Coculture-ASV15	0.041
ASV15-ASV4255	Plankton-Mono-ASV4255	Plankton-Coculture-ASV4255	0.320
ASV15-ASV4255	Biofilm-Mono-ASV15	Biofilm -Coculture-ASV15	0.413
ASV15-ASV4255	Biofilm -Mono-ASV4255	Biofilm -Coculture-ASV4255	0.018
ASV15-ASV4255	Total-Mono-ASV15	Total -Coculture-ASV15	0.033
ASV15-ASV4255	Total -Mono-ASV4255	Total -Coculture-ASV4255	0.021
ASV17-ASV11	Plankton-Mono-ASV17	Plankton-Coculture-ASV17	0.034
ASV17-ASV11	Plankton-Mono-ASV11	Plankton-Coculture-ASV11	0.443
ASV17-ASV11	Biofilm-Mono-ASV17	Biofilm -Coculture-ASV17	0.372
ASV17-ASV11	Biofilm -Mono-ASV11	Biofilm -Coculture-ASV11	0.013
ASV17-ASV11	Total-Mono-ASV17	Total -Coculture-ASV17	0.047
ASV17-ASV11	Total -Mono-ASV11	Total -Coculture-ASV11	0.012
ASV17-ASV198	Plankton-Mono-ASV17	Plankton-Coculture-ASV17	0.020
ASV17-ASV198	Plankton-Mono-ASV198	Plankton-Coculture-ASV198	0.109
ASV17-ASV198	Biofilm-Mono-ASV17	Biofilm -Coculture-ASV17	0.306
ASV17-ASV198	Biofilm -Mono-ASV198	Biofilm -Coculture-ASV198	0.008
ASV17-ASV198	Total-Mono-ASV17	Total -Coculture-ASV17	0.022
ASV17-ASV198	Total -Mono-ASV198	Total -Coculture-ASV198	0.049
ASV17-ASV404	Plankton-Mono-ASV17	Plankton-Coculture-ASV17	0.513
ASV17-ASV404	Plankton-Mono-ASV404	Plankton-Coculture-ASV404	0.275
ASV17-ASV404	Biofilm-Mono-ASV17	Biofilm -Coculture-ASV17	0.601
ASV17-ASV404	Biofilm -Mono-ASV404	Biofilm -Coculture-ASV404	0.056
ASV17-ASV404	Total-Mono-ASV17	Total -Coculture-ASV17	0.436
ASV17-ASV404	Total -Mono-ASV404	Total -Coculture-ASV404	0.092
ASV17-ASV3126	Plankton-Mono-ASV17	Plankton-Coculture-ASV17	0.028
ASV17-ASV3126	Plankton-Mono-ASV3126	Plankton-Coculture-ASV3126	0.266
ASV17-ASV3126	Biofilm-Mono-ASV17	Biofilm -Coculture-ASV17	0.367
ASV17-ASV3126	Biofilm -Mono-ASV3126	Biofilm -Coculture-ASV3126	0.001
ASV17-ASV3126	Total-Mono-ASV17	Total -Coculture-ASV17	0.027
ASV17-ASV3126	Total -Mono-ASV3126	Total -Coculture-ASV3126	0.001
ASV17-ASV4255	Plankton-Mono-ASV17	Plankton-Coculture-ASV17	0.062
ASV17-ASV4255	Plankton-Mono-ASV4255	Plankton-Coculture-ASV4255	0.051
ASV17-ASV4255	Biofilm-Mono-ASV17	Biofilm -Coculture-ASV17	0.525
ASV17-ASV4255	Biofilm -Mono-ASV4255	Biofilm -Coculture-ASV4255	0.044
ASV17-ASV4255	Total-Mono-ASV17	Total -Coculture-ASV17	0.041
ASV17-ASV4255	Total -Mono-ASV4255	Total -Coculture-ASV4255	0.047
ASV18-ASV11	Plankton-Mono-ASV18	Plankton-Coculture-ASV18	0.063
ASV18-ASV11	Plankton-Mono-ASV11	Plankton-Coculture-ASV11	0.453
ASV18-ASV11	Biofilm-Mono-ASV18	Biofilm -Coculture-ASV18	0.147
ASV18-ASV11	Biofilm -Mono-ASV11	Biofilm -Coculture-ASV11	0.046
ASV18-ASV11	Total-Mono-ASV18	Total -Coculture-ASV18	0.010
ASV18-ASV11	Total -Mono-ASV11	Total -Coculture-ASV11	0.047
ASV18-ASV198	Plankton-Mono-ASV18	Plankton-Coculture-ASV18	0.222

ASV18-ASV198	Plankton-Mono-ASV198	Plankton-Coculture-ASV198	0.152
ASV18-ASV198	Biofilm-Mono-ASV18	Biofilm -Coculture-ASV18	0.770
ASV18-ASV198	Biofilm -Mono-ASV198	Biofilm -Coculture-ASV198	0.375
ASV18-ASV198	Total-Mono-ASV18	Total -Coculture-ASV18	0.583
ASV18-ASV198	Total -Mono-ASV198	Total -Coculture-ASV198	0.220
ASV18-ASV404	Plankton-Mono-ASV18	Plankton-Coculture-ASV18	0.026
ASV18-ASV404	Plankton-Mono-ASV404	Plankton-Coculture-ASV404	0.613
ASV18-ASV404	Biofilm-Mono-ASV18	Biofilm -Coculture-ASV18	0.003
ASV18-ASV404	Biofilm -Mono-ASV404	Biofilm -Coculture-ASV404	0.888
ASV18-ASV404	Total-Mono-ASV18	Total -Coculture-ASV18	0.016
ASV18-ASV404	Total -Mono-ASV404	Total -Coculture-ASV404	0.784
ASV18-ASV3126	Plankton-Mono-ASV18	Plankton-Coculture-ASV18	0.850
ASV18-ASV3126	Plankton-Mono-ASV3126	Plankton-Coculture-ASV3126	0.618
ASV18-ASV3126	Biofilm-Mono-ASV18	Biofilm -Coculture-ASV18	0.672
ASV18-ASV3126	Biofilm -Mono-ASV3126	Biofilm -Coculture-ASV3126	0.462
ASV18-ASV3126	Total-Mono-ASV18	Total -Coculture-ASV18	0.628
ASV18-ASV3126	Total -Mono-ASV3126	Total -Coculture-ASV3126	0.640
ASV18-ASV4255	Plankton-Mono-ASV18	Plankton-Coculture-ASV18	0.719
ASV18-ASV4255	Plankton-Mono-ASV4255	Plankton-Coculture-ASV4255	0.889
ASV18-ASV4255	Biofilm-Mono-ASV18	Biofilm -Coculture-ASV18	0.350
ASV18-ASV4255	Biofilm -Mono-ASV4255	Biofilm -Coculture-ASV4255	0.002
ASV18-ASV4255	Total-Mono-ASV18	Total -Coculture-ASV18	0.332
ASV18-ASV4255	Total -Mono-ASV4255	Total -Coculture-ASV4255	4.639×10^{-4}

Supplementary Table 4. Statistical results of comparisons shown in Supplementary Figure 13.

The *p* value was determined by one-way ANOVA.

Isolate	Medium	Group 1	Group 2	F value	*p* value
ASV2	ISEM	Fresh medium	Conditioned by wild type	295.320	1.337×10^{-7}
ASV2	ISEM	Fresh medium	Conditioned by Δ*sfnaD*	37.885	2.725×10^{-4}
ASV2	ISEM	Conditioned by wild type	Conditioned by Δ*sfnaD*	7.644	0.024
ASV7	ISEM	Fresh medium	Conditioned by wild type	293.564	1.369×10^{-7}
ASV7	ISEM	Fresh medium	Conditioned by Δ*sfnaD*	24.991	0.001
ASV7	ISEM	Conditioned by wild type	Conditioned by Δ*sfnaD*	51.288	9.600×10^{-5}
ASV15	ISEM	Fresh medium	Conditioned by wild type	511.276	1.550×10^{-8}
ASV15	ISEM	Fresh medium	Conditioned by Δ*sfnaD*	22.069	0.002
ASV15	ISEM	Conditioned by wild type	Conditioned by Δ*sfnaD*	19.813	0.002
ASV17	ISEM	Fresh medium	Conditioned by wild type	164.491	1.289×10^{-6}
ASV17	ISEM	Fresh medium	Conditioned by Δ*sfnaD*	42.942	1.779×10^{-4}
ASV17	ISEM	Conditioned by wild type	Conditioned by Δ*sfnaD*	0.021	0.887
ASV18	ISEM	Fresh medium	Conditioned by wild type	256.461	2.318×10^{-7}
ASV18	ISEM	Fresh medium	Conditioned by Δ*sfnaD*	21.066	0.002
ASV18	ISEM	Conditioned by wild type	Conditioned by Δ*sfnaD*	28.598	0.001
ASV2	M9	Fresh medium	Conditioned by wild type	66.104	3.886×10^{-5}
ASV2	M9	Fresh medium	Conditioned by Δ*sfnaD*	9.265	0.016
ASV2	M9	Conditioned by wild type	Conditioned by Δ*sfnaD*	11.208	0.010
ASV7	M9	Fresh medium	Conditioned by wild type	69.590	3.227×10^{-5}
ASV7	M9	Fresh medium	Conditioned by Δ*sfnaD*	295.157	1.340×10^{-7}
ASV7	M9	Conditioned by wild type	Conditioned by Δ*sfnaD*	2.769	0.135
ASV15	M9	Fresh medium	Conditioned by wild type	0.320	0.587
ASV15	M9	Fresh medium	Conditioned by Δ*sfnaD*	0.265	0.620
ASV15	M9	Conditioned by wild type	Conditioned by Δ*sfnaD*	2.627	0.144
ASV17	M9	Fresh medium	Conditioned by wild type	39.968	2.273×10^{-4}
ASV17	M9	Fresh medium	Conditioned by Δ*sfnaD*	14.921	0.005
ASV17	M9	Conditioned by wild type	Conditioned by Δ*sfnaD*	7.425	0.026
ASV18	M9	Fresh medium	Conditioned by wild type	21.306	0.002
ASV18	M9	Fresh medium	Conditioned by Δ*sfnaD*	9.667	0.014

ASV18	M9	Conditioned by wild type	Conditioned by Δ*sfnaD*	6.414	0.035

Supplementary Table 5. Statistical results of comparisons shown in Figure 5b. The *p* value was determined by one-way ANOVA.

Genus	DAA level 1 (mg/L)	DAA level 2 (mg/L)	F value	*p* value
Pseudomonas	25	35	17.383	2.664×10^{-4}
Pseudomonas	25	45	111.718	2.794×10^{-11}
Pseudomonas	25	55	243.994	2.359×10^{-15}
Pseudomonas	35	45	49.414	1.205×10^{-7}
Pseudomonas	35	55	159.865	4.299×10^{-13}
Pseudomonas	45	55	34.909	2.342×10^{-6}
Rhodococcus	25	35	20.995	8.688×10^{-5}
Rhodococcus	25	45	65.495	8.221×10^{-9}
Rhodococcus	25	55	137.196	2.631×10^{-12}
Rhodococcus	35	45	20.980	8.729×10^{-5}
Rhodococcus	35	55	90.555	2.844×10^{-10}
Rhodococcus	45	55	39.229	9.038×10^{-7}

Supplementary Table 6. One-way ANOVA test of the proportion of *Pseudomonas* cells inhabiting biofilms in monoculture (mean 0.585, standard deviation 0.128, n = 5 isolates × 4 replicates) versus that in co-culture (mean 0.809, standard deviation 0.122, n = 25 combinations × 4 replicates).

Degrees of freedom	Sums of squares	Mean squares	F value	*p* value
1	0.8388	0.8388	55.15	1.91×10^{-11}

Supplementary Table 7. Statistical results of comparisons shown in Supplementary Figure 19.

The *p* value was calculated using one-way ANOVA.

Isolate	medium	DAA level 1 (mg/L)	DAA level 2 (mg/L)	F value	*p* value
ASV2	ISEM	15	25	1.264	0.324
ASV2	ISEM	15	45	0.701	0.450
ASV2	ISEM	15	65	0.425	0.550
ASV2	ISEM	25	45	0.138	0.729
ASV2	ISEM	25	65	0.237	0.652
ASV2	ISEM	45	65	0.026	0.879
ASV15	ISEM	15	25	1.738	0.258
ASV15	ISEM	15	45	0.002	0.965
ASV15	ISEM	15	65	0.187	0.688
ASV15	ISEM	25	45	1.212	0.333
ASV15	ISEM	25	65	2.248	0.208
ASV15	ISEM	45	65	0.112	0.755
ASV18	ISEM	15	25	3.375×10^{-5}	0.996
ASV18	ISEM	15	45	0.022	0.890
ASV18	ISEM	15	65	0.129	0.738
ASV18	ISEM	25	45	0.024	0.884
ASV18	ISEM	25	65	0.132	0.734
ASV18	ISEM	45	65	0.071	0.802
ASV17	ISEM	15	25	0.524	0.509
ASV17	ISEM	15	45	0.106	0.762
ASV17	ISEM	15	65	3.082	0.154
ASV17	ISEM	25	45	0.054	0.828
ASV17	ISEM	25	65	3.485	0.135
ASV17	ISEM	45	65	1.585	0.277
ASV7	ISEM	15	25	7.912	0.048
ASV7	ISEM	15	45	20.894	0.010
ASV7	ISEM	15	65	17.097	0.014
ASV7	ISEM	25	45	4.152	0.111
ASV7	ISEM	25	65	2.001	0.230
ASV7	ISEM	45	65	0.489	0.523
ASV2	M9	0	15	1.875	0.243
ASV2	M9	0	25	11.319	0.028
ASV2	M9	0	45	19.064	0.012
ASV2	M9	0	65	17.572	0.014
ASV2	M9	15	25	9.381	0.038
ASV2	M9	15	45	19.458	0.012
ASV2	M9	15	65	15.341	0.017
ASV2	M9	25	45	5.045	0.088

ASV2	M9	25	65	4.407	0.104
ASV2	M9	45	65	0.179	0.694
ASV15	M9	0	15	0.432	0.547
ASV15	M9	0	25	1.553	0.281
ASV15	M9	0	45	48.204	0.002
ASV15	M9	0	65	102.056	0.001
ASV15	M9	15	25	2.911	0.163
ASV15	M9	15	45	87.454	0.001
ASV15	M9	15	65	169.947	1.998×10^{-4}
ASV15	M9	25	45	7.212	0.055
ASV15	M9	25	65	21.413	0.010
ASV15	M9	45	65	19.363	0.012
ASV18	M9	0	15	8.752	0.042
ASV18	M9	0	25	9.468	0.037
ASV18	M9	0	45	29.707	0.006
ASV18	M9	0	65	52.604	0.002
ASV18	M9	15	25	0.135	0.732
ASV18	M9	15	45	9.900	0.035
ASV18	M9	15	65	26.376	0.007
ASV18	M9	25	45	6.578	0.062
ASV18	M9	25	65	18.091	0.013
ASV18	M9	45	65	1.903	0.240
ASV17	M9	0	15	2.477	0.191
ASV17	M9	0	25	17.864	0.013
ASV17	M9	0	45	24.085	0.008
ASV17	M9	0	65	136.527	3.068×10^{-4}
ASV17	M9	15	25	45.311	0.003
ASV17	M9	15	45	52.057	0.002
ASV17	M9	15	65	783.519	9.691×10^{-6}
ASV17	M9	25	45	0.872	0.403
ASV17	M9	25	65	24.885	0.008
ASV17	M9	45	65	10.216	0.033
ASV7	M9	0	15	5.867	0.073
ASV7	M9	0	25	12.830	0.023
ASV7	M9	0	45	66.961	0.001
ASV7	M9	0	65	191.053	1.588×10^{-4}
ASV7	M9	15	25	2.849	0.167
ASV7	M9	15	45	36.415	0.004
ASV7	M9	15	65	144.987	2.728×10^{-4}
ASV7	M9	25	45	8.433	0.044
ASV7	M9	25	65	52.532	0.002
ASV7	M9	45	65	37.435	0.004

Supplementary Table 8. Calibration curve precision and accuracy for amino acid quantification

Amino acid	Theoretical concentration (mg/L)	RSD (%)	Measured concentration (mg/L)	Bias (%)
L-Thr	1	8.08	0.9450	-5.50
L-Thr	4	3.18	4.0879	2.20
L-Thr	9	2.38	8.9669	-0.37
D-Thr	1	1.99	1.1138	11.38
D-Thr	4	1.21	3.8181	-4.55
D-Thr	9	0.95	9.0684	0.76
L-Val	1	1.99	1.0529	5.29
L-Val	4	4.17	3.9154	-2.12
L-Val	9	2.62	9.0319	0.35
D-Val	1	1.10	1.0055	0.55
D-Val	4	0.81	3.9911	-0.22
D-Val	9	1.75	9.0033	0.04
L-Met	1	12.42	0.9375	-6.25
L-Met	4	5.83	4.1001	2.50
L-Met	9	4.08	8.9626	-0.42
D-Met	1	3.56	1.1040	10.40
D-Met	4	0.98	3.8337	-4.16
D-Met	9	2.44	9.0625	0.69
L-Leu	1	10.81	0.9771	-2.29
L-Leu	4	5.23	4.0367	0.92
L-Leu	9	1.86	8.9862	-0.15
D-Leu	1	5.39	0.9187	-8.13
D-Leu	4	2.21	4.1302	3.25
D-Leu	9	0.77	8.9513	-0.54
L-Phe	1	0.68	1.0680	6.80
L-Phe	4	2.72	3.8913	-2.72
L-Phe	9	9.35	9.0407	0.45
D-Phe	1	5.83	0.9950	-0.50
D-Phe	4	1.68	4.0081	0.20

D-Phe	9	0.25	8.9972	-0.03
L-His	1	2.00	1.0347	3.47
L-His	4	5.04	3.9446	-1.39
L-His	9	3.43	9.0210	0.23
D-His	1	13.62	1.0035	0.35
D-His	4	1.21	3.9944	-0.14
D-His	9	2.99	9.0020	0.02
L-Lys	1	7.64	1.1363	13.63
L-Lys	4	4.13	3.7819	-5.45
L-Lys	9	2.94	9.0817	0.91
D-Lys	1	6.98	1.0052	0.53
D-Lys	4	4.79	3.9917	-0.21
D-Lys	9	6.36	9.0033	0.04
L-Trp	1	3.57	0.9967	-0.33
L-Trp	4	4.72	4.0054	0.14
L-Trp	9	1.03	8.9981	-0.02
D-Trp	1	7.37	1.0490	4.90
D-Trp	4	3.03	3.9216	-1.96
D-Trp	9	2.76	9.0294	0.33

Supplementary Table 9. Primers used to construct siderophore synthesis mutant

Primer name	Description	Sequence
sidupS	Upstream homologous arm amplification	GCGATATCGAGCTCGACGCCTATCGTGGGTTTG
sidupA		GTAACAACCGATTCAAAGGGCTTCGCTCCAGTGT
siddwS	Downstream homologous arm amplification	TTGAATCGGTTGTTACTGCG
siddwA		TCCCGGGAGAGCTCAAGGTGTAGGCCGGTTGC
Ins-sfnaD-S	Inside primers specific to the upstream and downstream region of *sfnaD*	GCGAATACGACGACAACCG
Ins-sfnaD-A		TCGAGAATATCCAGCAACAGCT
Out-sfnaD-S	Outside primers specific to the upstream and downstream region of *sfnaD*	CGCAGAGGAAGGTGAAGAAGG
Out-sfnaD-A		AGGAGCCAAGCAGCGGAAT

Supplementary Table 10. Calibration curve precision and accuracy for flavin quantification

Flavin	Theoretical concentration (mg/L)	RSD (%)	Measured concentration (mg/L)	Bias (%)
RF	0.4	2.78	0.40	0.06
RF	0.8	2.48	0.72	-10.1
RF	2.0	8.86	1.99	-0.51
FMN	0.4	0.61	0.46	14.62
FMN	0.8	0.80	0.84	5.58
FMN	2.0	7.07	1.97	-1.45
FAD	0.4	5.53	0.40	-0.31
FAD	0.8	2.96	0.80	-0.44
FAD	2.0	4.22	2.0	0.05
