# Supplementary material for: Cooperative microbial interactions drive spatial segregation in porous environments
Source: Nat Commun. 2023 Jul 15;14:4226. doi: 10.1038/s41467-023-39991-4 (PMC10349867; doi:10.1038/s41467-023-39991-4)
Supplement: Supplementary file 3 — Reporting Summary [file 41467_2023_39991_MOESM3_ESM.pdf]

nature portfolio

Corresponding author(s): Peng Cai

Last updated by author(s): Jun 16, 2023

Reporting Summary

Nature Portfolio wishes to improve the reproducibility of the work that we publish. This form provides structure for consistency and transparency in reporting. For further information on Nature Portfolio policies, see our Editorial Policies and the Editorial Policy Checklist.

Statistics

For all statistical analyses, confirm that the following items are present in the figure legend, table legend, main text, or Methods section.

n/a	Confirmed	
☐	☒	The exact sample size (n) for each experimental group/condition, given as a discrete number and unit of measurement
☐	☒	A statement on whether measurements were taken from distinct samples or whether the same sample was measured repeatedly
☐	☒	The statistical test(s) used AND whether they are one- or two-sided<br>*Only common tests should be described solely by name; describe more complex techniques in the Methods section.*
☒	☐	A description of all covariates tested
☒	☐	A description of any assumptions or corrections, such as tests of normality and adjustment for multiple comparisons
☐	☒	A full description of the statistical parameters including central tendency (e.g. means) or other basic estimates (e.g. regression coefficient) AND variation (e.g. standard deviation) or associated estimates of uncertainty (e.g. confidence intervals)
☐	☒	For null hypothesis testing, the test statistic (e.g. F, t, r) with confidence intervals, effect sizes, degrees of freedom and P value noted<br>*Give P values as exact values whenever suitable.*
☒	☐	For Bayesian analysis, information on the choice of priors and Markov chain Monte Carlo settings
☒	☐	For hierarchical and complex designs, identification of the appropriate level for tests and full reporting of outcomes
☐	☒	Estimates of effect sizes (e.g. Cohen's d, Pearson's r), indicating how they were calculated

Our web collection on statistics for biologists contains articles on many of the points above.

Software and code

Policy information about availability of computer code

Data collection	not applicable
Data analysis	Confocal images were analysed by a custom-written MATLAB code which has been stored in the figshare database (https://figshare.com/projects/Cooperative_interactions_drive_spatial_segregation/156834). The raw sequencing data was processed using the DADA2 (version 2021.2.0) in QIIME2 (version 2021.2) to merge and denoise paired-end reads. Taxonomy was assigned using Naive Bayes classifier (version 2021.2.0). The community assembly mechanisms were quantitatively inferred by a phylogenetic-bin-based null model analysis (iCAMP) using package “iCAMP” (version 1.3.4). The raw MS data were converted to mzXML format using MSConvert (version 3.0) and were subsequently processed by XCMS (version 3.2) for peak picking and alignment. For genomic analysis, quality filtering and adaptor removal were carried out using AdapterRemoval (version 2.2.2). The filtered reads were assembled via A5-MiSeq (version 20160825) and SPAdes (version 3.12.0). For transcriptomic analysis, Htseq-count (version 1.6.0) was used to generate the read count of individual gene and the differentially expressed genes (fold change ≥ 2, p-value < 0.05) were identified by DESeq2 (version 1.28.1). KEGG functional enrichment analysis was performed using ClusterProfiler (version 4.0.0).

For manuscripts utilizing custom algorithms or software that are central to the research but not yet described in published literature, software must be made available to editors and reviewers. We strongly encourage code deposition in a community repository (e.g. GitHub). See the Nature Portfolio guidelines for submitting code & software for further information.

Data

Policy information about availability of data

All manuscripts must include a data availability statement. This statement should provide the following information, where applicable:

- Accession codes, unique identifiers, or web links for publicly available datasets
- A description of any restrictions on data availability
- For clinical datasets or third party data, please ensure that the statement adheres to our policy

Source data are provided with this paper. The raw amplicon and transcriptomic sequencing data are deposited in the NCBI SRA database with the accession number of PRJNA764456 (https://www.ncbi.nlm.nih.gov/sra?linkname=bioproject_sra_all&from_uid=764456) and PRJNA813193 (https://www.ncbi.nlm.nih.gov/sra/?term=813193). The reference libraries for metabolite identification are available from METLIN (http://metlin.scripps.edu), MassBank (https://massbank.eu/MassBank/), LipidMaps (https://www.lipidmaps.org/), and mzCloud (https://www.mzcloud.org/). The taxonomy reference is available at the Greengenes database (http://greengenes.lbl.gov). The KEGG pathways can be accessed through the KEGG database (https://www.genome.jp/kegg/). The genomic data for bacterial isolates is available in the IMG database (https://img.jgi.doe.gov/). Raw data including metabolomics data, confocal images of biofilm during colonization, dynamics of community composition, detected metabolites in metabolomics, differentially expressed genes in transcriptomic data, biofilm yield and planktonic growth in monoculture and co-culture, abundances of different genotypes in co-culture and concentrations of AAs during biofilm development have been deposited in the figshare database (https://figshare.com/projects/Cooperative_interactions_drive_spatial_segregation/156834).

Research involving human participants, their data, or biological material

Policy information about studies with human participants or human data. See also policy information about sex, gender (identity/presentation), and sexual orientation and race, ethnicity and racism.

Reporting on sex and gender	not applicable
Reporting on race, ethnicity, or other socially relevant groupings	not applicable
Population characteristics	not applicable
Recruitment	not applicable
Ethics oversight	not applicable

Note that full information on the approval of the study protocol must also be provided in the manuscript.

Field-specific reporting

Please select the one below that is the best fit for your research. If you are not sure, read the appropriate sections before making your selection.

☒ Life sciences ☐ Behavioural & social sciences ☐ Ecological, evolutionary & environmental sciences

For a reference copy of the document with all sections, see nature.com/documents/nr-reporting-summary-flat.pdf

Life sciences study design

All studies must disclose on these points even when the disclosure is negative.

Sample size	We employed 25 independent microfluidic chambers to cultivate biofilms simultaneously to analyze the microbial assembly processes. At each sampling point, we randomly selected six chambers to collect effluents for metabolomic analysis and three chambers for the destructive collection of the total microbial communities. This approach facilitated the evaluation of biological variability of the community composition and ensured a manageable experiment. For FISH and microscopic characterization, we employed four independent chambers for each time point and randomly selected 15 grains from each replicate to capture images of the biofilm structure, ensuring a sufficient analysis of variability in biofilm structure. We conducted three biological replicates in the transcriptomic analysis, enabling statistical analyses to evaluate the significance of gene expression changes while considering cost considerations. Eight biological replicates were conducted in the microbial interaction analyses, providing a substantial number of replicates for analyzing the variability in planktonic and biofilm growth.<br>To assess the capability of DAA hydrolysis and the influence of DAA on biofilm formation, we conducted three biological replicates for each of the five different isolates within each genus, providing an ample sample size to analyze the variability in DAA hydrolysis capability and biofilm formation within a genus.
Data exclusions	We excluded microfluidic chambers from the analysis based on the following criteria: 1) obvious problems with the microfluidic setup, such as a stalled syringe pump or air bubbles in the chambers, and 2) segmentation issues when image quality was insufficient to ensure proper segmentation with our algorithm.
Replication	Each condition/group underwent at least three biological replicates and similar results were obtained.
Randomization	For the biofilm development experiments, we randomly selected three microfluidic chips at each time point in the time-series to collect biofilm samples for subsequent analysis. For the microbial interaction analysis, we randomly assigned a subset of cells from the same bacterial

	culture to the experimental conditions.
Blinding	The study primarily focused on biofilm structure and microbial community during biofilm development and the influence of microbial interaction. Therefore, blinding was not applicable in this context.

Reporting for specific materials, systems and methods

We require information from authors about some types of materials, experimental systems and methods used in many studies. Here, indicate whether each material, system or method listed is relevant to your study. If you are not sure if a list item applies to your research, read the appropriate section before selecting a response.

Materials & experimental systems

n/a	Involved in the study
☒	☐ Antibodies
☒	☐ Eukaryotic cell lines
☒	☐ Palaeontology and archaeology
☒	☐ Animals and other organisms
☒	☐ Clinical data
☒	☐ Dual use research of concern
☒	☐ Plants

Methods

n/a	Involved in the study
☒	☐ ChIP-seq
☒	☐ Flow cytometry
☒	☐ MRI-based neuroimaging
